# AUD-DSS: a decision support system for early detection of patients with alcohol use disorder

**DOI:** 10.1186/s12859-023-05450-6

**Published:** 2023-09-02

**Authors:** Ali Ebrahimi, Uffe Kock Wiil, Ruben Baskaran, Abdolrahman Peimankar, Kjeld Andersen, Anette Søgaard Nielsen

**Affiliations:** 1 SDU Health Informatics and Technology, The Maersk Mc-Kinney Moller Institute, University of Southern Denmark, Odense, Denmark; 2 Unit for Clinical Alcohol Research, Clinical Institute, University of Southern Denmark, Odense, Denmark

**Keywords:** Alcohol use disorder, Machine learning, Stacking ensemble, Feature selection, Imbalanced data

## Abstract

**Background:**

Alcohol use disorder (AUD) causes significant morbidity, mortality, and injuries. According to reports, approximately 5% of all registered deaths in Denmark could be due to AUD. The problem is compounded by the late identification of patients with AUD, a situation that can cause enormous problems, from psychological to physical to economic problems. Many individuals suffering from AUD never undergo specialist treatment during their addiction due to obstacles such as taboo and the poor performance of current screening tools. Therefore, there is a lack of rapid intervention. This can be mitigated by the early detection of patients with AUD. A clinical decision support system (DSS) powered by machine learning (ML) methods can be used to diagnose patients’ AUD status earlier.

**Methods:**

This study proposes an effective AUD prediction model (AUDPM), which can be used in a DSS. The proposed model consists of four distinct components: (1) imputation to address missing values using the k-nearest neighbours approach, (2) recursive feature elimination with cross validation to select the most relevant subset of features, (3) a hybrid synthetic minority oversampling technique-edited nearest neighbour approach to remove noise and balance the distribution of the training data, and (4) an ML model for the early detection of patients with AUD.

Two data sources, including a questionnaire and electronic health records of 2571 patients, were collected from Odense University Hospital in the Region of Southern Denmark for the AUD-Dataset. Then, the AUD-Dataset was used to build ML models. The results of different ML models, such as support vector machine, K-nearest neighbour, decision tree, random forest, and extreme gradient boosting, were compared. Finally, a combination of all these models in an ensemble learning approach was selected for the AUDPM.

**Results:**

The results revealed that the proposed ensemble AUDPM outperformed other single models and our previous study results, achieving 0.96, 0.94, 0.95, and 0.97 precision, recall, F1-score, and accuracy, respectively. In addition, we designed and developed an AUD-DSS prototype.

**Conclusion:**

It was shown that our proposed AUDPM achieved high classification performance. In addition, we identified clinical factors related to the early detection of patients with AUD. The designed AUD-DSS is intended to be integrated into the existing Danish health care system to provide novel information to clinical staff if a patient shows signs of harmful alcohol use; in other words, it gives staff a good reason for having a conversation with patients for whom a conversation is relevant.

**Supplementary Information:**

The online version contains supplementary material available at 10.1186/s12859-023-05450-6.

## Introduction

Alcohol use disorder (AUD) is a diagnostic term used to refer to the problematic use of alcohol. According to the DSM-5 [1], a person diagnosed with AUD exhibits a *“problematic pattern of alcohol use leading to clinically significant impairment or distress”.* It is one of the most common worldwide occurrences of all psychiatric disorders [2]. AUD is a serious, painful, prevalent, and costly affliction. Cross-sectional studies conducted in the Nordic region have shown that Finland had the highest prevalence of harm resulting from AUD, with a total prevalence of 53% within the past 12 months, followed by Denmark at 44%, Sweden at 38%, and Norway at the lowest prevalence of 25% [3, 4]. In the United States, AUD claims the lives of 88,000 people each year [5, 6]. According to reports, approximately 5% of all registered deaths in Denmark could be due to AUD, a situation that is common in most Western countries [7–10].

Despite the apparent issues caused by AUD, it remains one of the most undertreated disorders. In a major survey involving 13,000 patients and 358 general practitioners across six European countries, only 22.3% of patients who were diagnosed with alcohol dependence received treatment [11]. In Denmark, it was estimated that there were 585,000 people with hazardous alcohol use, with 140,000 people suffering from alcohol dependence [8]. However, only 15,000 [12] of them had sought specialist treatment for their alcohol problem, and this often occurs after more than a decade of suffering from AUD [13].

Individuals with problematic alcohol use can be identified according to a set of standard criteria designed for AUD. There are three methods based on the criteria for screening and diagnosis in 2009 [14], through a blood test, clinical course, or questionnaire. For the identification of AUD, routine screening that combines one or two screening methods has been recommended. However, it can be difficult to identify individuals with AUD because symptoms of AUD are often not obvious and visible. The symptoms individuals with AUD present are often general symptoms present in other medical conditions and not specific for AUD (lethargy, anxiety, insomnia, etc.).

At hospitals, there are many barriers to the systematic screening of AUD and the rapid intervention approaches developed for AUD patients, such as the heavy workload of staff, fear of patient confrontation, inadequate preparation, and need to focus on a particular medical condition in highly specialized departments [15–17]. The taboo and stigma associated with harmful drinking may be some of the reasons for the considerably late identification of AUD.

Patients with AUD are highly prevalent in the health care system. According to a study by Oxholm et al. [16], patients expressed a willingness to discuss lifestyle issues, providing an opportunity for health care professionals to address these concerns. However, there may be significant delays in both diagnosis and treatment in actual practice. Carvalho et al. [18] identified three main contributors to this gap: individual-level factors, clinicians, and the absence of a formal screening process. The inadequate performance of conventional AUD screening methods and the lack of a systematic screening procedure are believed to be the primary causes of the late identification of AUD. To bridge this gap, we aim to investigate the utilization of electronic health records (EHRs) and machine learning (ML) algorithms to develop predictive models for the early detection of patients with AUD to offer a solution to this issue.

Although highly monitored trials have shown that primary health workers can positively detect patients’ drinking levels [19] as well as make referrals for AUD treatment, their effectiveness seems to have little impact since there is a lack of intelligent clinical screening methods. The recent availability of vast amounts of EHR data and the advancement of ML algorithms have made it possible to offer reasoning for clinical staff to support their decisions. ML has emerged as a promising approach for clinical decision support tools across various health care domains. ML techniques have been successfully employed in diverse areas, including the detection of dementia and Alzheimer’s disease [20, 21], early detection of diabetes [22], detection of atrial fibrillation [23], as well as the early detection of AUD, among others.

Previous studies have shown that ML algorithms such as artificial neural networks [24–26], logistic regression (LR) [25, 27–29], support vector machines (SVMs) [24–26, 29–32], random forests (RFs) [24, 25, 28, 29, 31], elastic nets [24, 31], k-nearest neighbour (KNN) [25], decision trees (DTs) [25, 28], and naive Bayes [28]

Previous studies have shown that ML algorithms such as artificial neural networks [24–26], logistic regression (LR) [25, 27–29], support vector machines (SVM) [24–26, 29–32], random forests (RF) [24, 25, 28, 29, 31], elastic nets [24, 31], k-nearest neighbour (KNN) [25], decision trees (DT) [25, 28], naive bayes [28], etc., have been successfully utilized to develop predictive models for the early detection of patients with AUD based on EHRs. However, in the ML field, missing values, feature redundancy, noisy datasets, and imbalanced data may arise and impact the performance of such prediction models [33, 34]. Previous studies have reported that by addressing the feature redundancy and imbalanced class distribution problems and handling missing values, the predictive performance of ML models can be enhanced significantly [35, 36]. However, previous studies in the early detection of AUD did not address all of these challenges in one pipeline. To the best of our knowledge, this is the first comprehensive study to implement a predictive model for AUD (AUDPM) that handles missing values, eliminates noise, selects the best subset of features, and balances class distribution from patients' EHRs. Moreover, there have been no previous studies on the development of a real-life ML-based clinical decision support system (DSS) for the detection of patients with AUD.

To conduct this study, two sources of data were collected from patients who were admitted to Odense University Hospital (OUH) and were used to develop the AUDPM, which is based on the stacking ensemble (SE) technique. The performance of the developed AUDPM was compared with that of other developed models, such as SVM, KNN, DT, RF, and XGBoost. In addition, we ensured the applicability of the proposed model by designing and implementing it into an AUD decision support system (AUD-DSS) for the early detection of AUD in patients based on their EHRs. The developed AUD-DSS is expected to help clinicians diagnose patients effectively and efficiently, thereby improving AUD clinical decision-making. Therefore, early intervention could be conducted to prevent the deaths caused by late AUD diagnosis. The contributions of our study can be summarized as follows:

1. *Improving the performance of an AUD predictive model* We developed the AUDPM by integrating the KNNImputer method to impute missing values, RFE feature selection method to reduce the high dimensionality problem, SMOTE-ENN to remove noise and balance the dataset, and an ML algorithm to improve the prediction accuracy. The AUDPM was trained based on a historical multidimensional database and developed for the early detection of patients' AUD status based on their historical EHRs.
2. *Analysing the model performance and comparing it with that of state-of-the-art predictive models* The efficacy of the proposed AUDPM was evaluated by comparing it to baseline models developed using an imbalanced dataset containing complete features and missing values. Furthermore, we compared the results of our model with those of previous studies. Our analysis not only highlights the significance of our model compared to other existing models but also includes a statistical evaluation.
3. *Identifying clinical factors* The most important clinical factors related to the early detection of patients with AUD based on EHRs are extracted from the developed ML algorithms and presented based on their importance.
4. *Developing a real-world system* To demonstrate the viability and usability of our suggested model for a real-world case study, we developed a prototype of the system. It is anticipated that the proposed system will serve as a useful reference for clinical staff.

The remainder of this paper is organized as follows. The proposed AUDPM, including the characteristics of the historical EHR dataset, the proposed framework, and the experimental setup that is used to evaluate the performance of the developed predictive models, is presented in Section two. Sections three presents the results. Section four present discuss the experimental results, a comparison to previous studies, limitations, and future works. The conclusion is presented in Section five.

## Methods

**Fig. 1:**
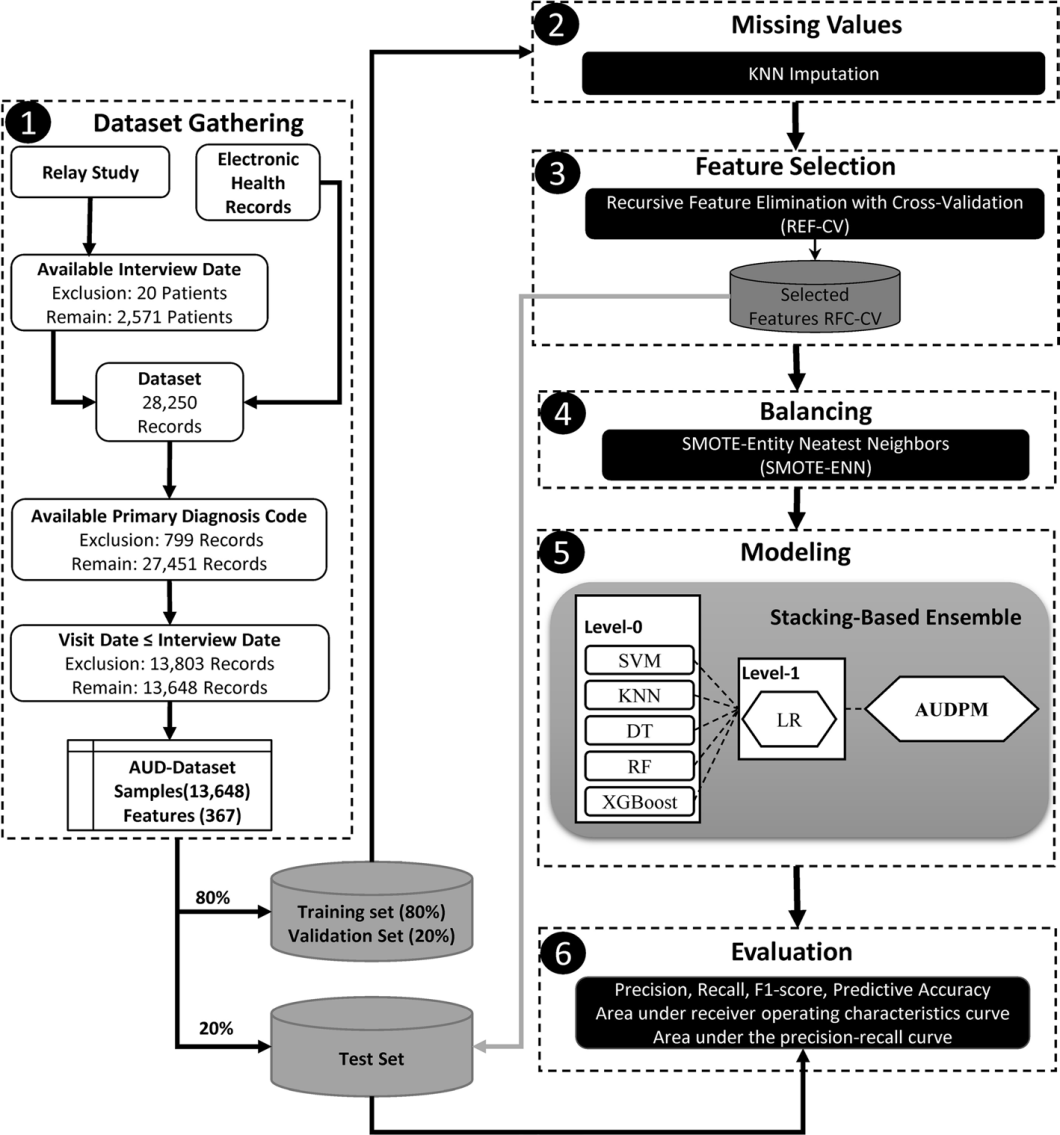
Proposed method

As shown in Fig. 1, the overall methodology proposed for this study encompasses four phases: 1) gathering data, 2) imputing missing values, 3) selecting features, 4) handling imbalanced class distribution, 5) developing the model, and 6) evaluating the model. In this study, clinical researchers were engaged through all stages of the proposed methodology. For example, in addition to storing datasets in a secure database, they declared the main idea of labelling patients’ EHRs based on the results of the Relay study [37–39]. Moreover, medical reasoning about individual clinical factors, specifically the primary diagnosis, was discussed in detail with them over several iterations. The TRIPOD checklist [40] can be found in the Additional file 1.

### AUD dataset

**Fig. 2:**
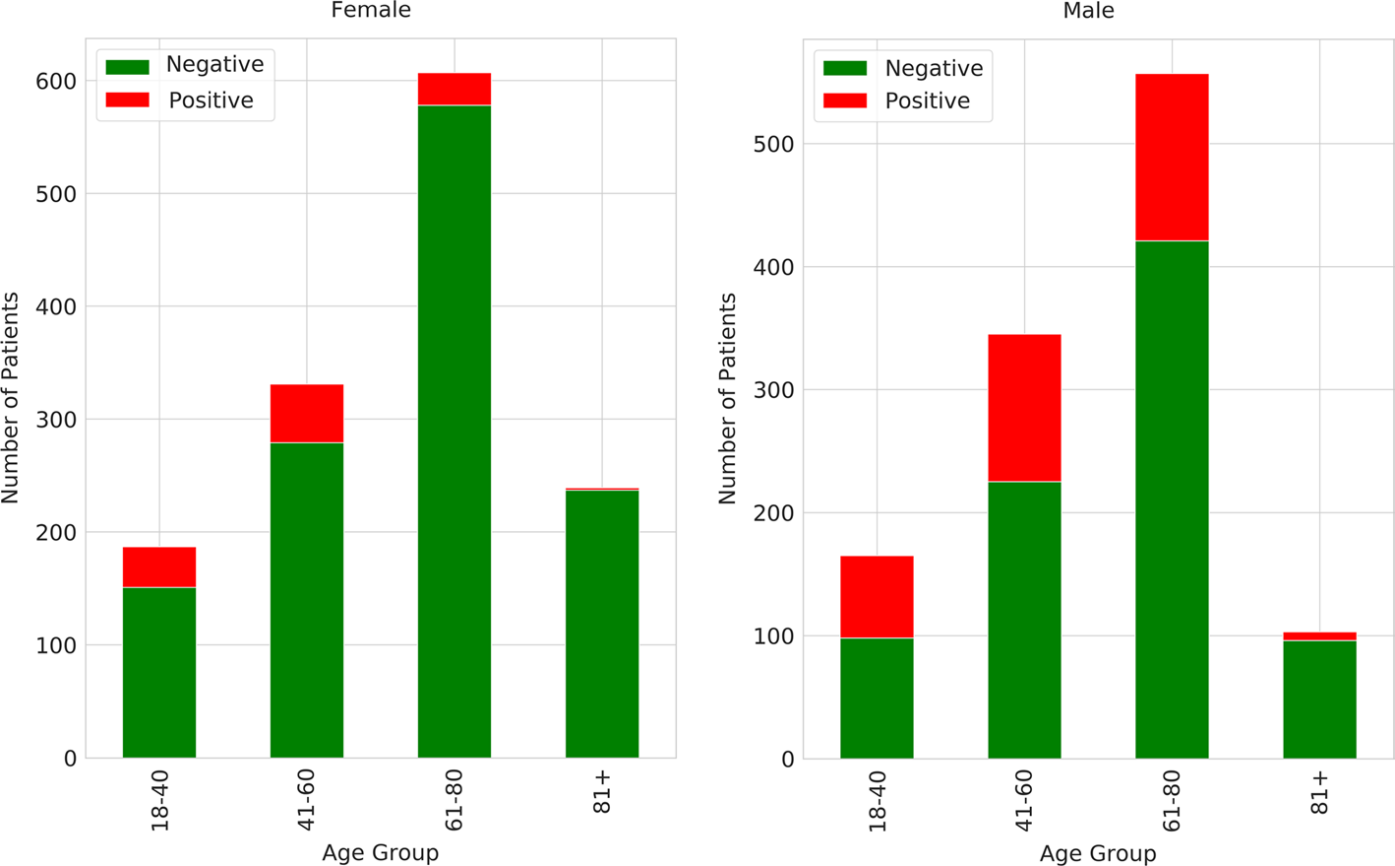
Distribution of patients based on their age and gender

The study cohort comprised 2551 individuals ranging in age from 18 to 101 years, all of whom were admitted to the OUH in Denmark for a minimum duration of 24 h spanning from January 2012 to June 2016. The data utilized in the study emanated from two primary sources, namely the Relay Project and the EHRs sourced from OUH. During the period spanning from October 2013 to June 2016, the Relay Project meticulously collected data from patients who underwent hospitalization within OUH's Gastrointestinal, Neurological, and Orthopaedic Departments. Through participation in a survey grounded in the Danish iteration of the Alcohol Use Disorder Identification Test (AUDIT) [41, 42], patients documented pertinent information regarding their dietary patterns, tobacco consumption, alcohol intake, and physical activity routines. The framework of the Relay Study was constructed upon a version of the AUDIT questionnaire tailored to the Danish context [42, 43], which yielded scores between 0 and 40 based on the patients’ responses. As per the threshold criteria established within the AUDIT assessment, the scores can serve to categorize individuals into either AUD-Negative or AUD-Positive groups. Patients with scores of 0–8 were classified as AUD-Negative while those with scores of 8–40 were classified as AUD-Positive. Therefore, in this study, AUDIT test scores were used to label the EHRs. Based on the results of the AUDIT test, among all the patients, 2096 patients were “AUD-Positive” and 455 patients were “AUD-Negative”. This categorization was used to label the collected EHR data from OUH as the target value for training the predictive models. Illustrated in Fig. 2 is the segmentation of the gathered data based on gender, age bracket, and AUD classification (Table 1).

The EHR dataset encompasses 13,648 clinical records pertaining to patients involved in the Relay study. This dataset encompasses individual-specific attributes such as the national identification number (CPR number) in Denmark, age, gender, duration of hospital sojourns, manner of admission, diagnostic codes denoted by ICD-10 nomenclature, and health-related ailments spanning a period of 18 months prior to OUH admission up to engagement in the Relay interview. Based on each person’s social security number, their EHR and Relay Study records were linked. For data security purposes and to comply with the GDPR, all social security numbers were anonymized. The final dataset was then stored on secure virtual servers run by the Open Patient Data Explorative Network in the Region of Southern Denmark. Based on the AUDIT test performance, clinical records were labelled "AUD-Positive" or "AUD-Negative". The final dataset is referred to as the *AUD-Dataset* in the subsequent descriptions. Table 1 shows the list and definitions of variables in the AUD-Dataset. Figure 1 shows more information about the AUD-Dataset's inclusion and exclusion criteria. More information about the distribution of features can be found in the Additional file 2.

### Missing value imputation

Missing values in datasets create significant analytical challenges in health care prediction. As shown in Table 1, the AUD-Dataset contains missing values in some features. Missing values in the AUD-Dataset may reduce the power/fitness of a classifier or lead to a biased classifier since the behaviour and connection between other features have not been sufficiently assessed. Many ML algorithms require imputation of these missing attribute values before proceeding. In this work, KNNImputer is utilized to replace the missing values. KNNImputer is a very accurate nonparametric method that finds the closest k-neighbours to a missing point in the multidimensional space [44].

**Table 1:** AUD-dataset description

Variable	Description	Missing ratio (%)		*P*-value	Feature range
		AUD-positive	AUD-negative		
AUD status	AUD-Positive / AUD-Negative			–	–
Gender	Male or Female	0	0	< 0.05	$\left({f}_{1},{f}_{2}\right)$
Age	Age of patient at time of Relay study	0	0	< 0.05	$\left({f}_{3}\right)$
Admission type	Admitted patients or outpatients	0	0	< 0.05	$\left({f}_{4},{f}_{5}\right)$
LOS	The amount of time the patient spent at the hospital for each visit	0	0	< 0.05	$\left({f}_{6}\right)$
ED	If the patient visited the emergency department prior to admission	0	0	< 0.05	$\left({f}_{7},{f}_{8}\right)$
ICU	If the patient was transferred to the ICU	0	0	< 0.05	$\left({f}_{9},{f}_{10}\right)$
Action Diagnosis	Reason why patients visited the hospital, scored according to the Danish version of ICD10 codes	0	0	–	$\left({f}_{11},{f}_{12},\dots ,{f}_{361}\right)$
DBP	Diastolic Blood pressure	4	22	< 0.05	$\left({f}_{362}\right)$
SBP	Systolic Blood pressure	4	22	< 0.05	$\left({f}_{363}\right)$
SaO2	Oxygen saturation	6	37	0.06	$\left({f}_{364}\right)$
Temp	Body temperature	8	50	0.99	$\left({f}_{365}\right)$
BMI	Body mass index	9	41	< 0.05	$\left({f}_{366}\right)$
Weight	Weight of patients	9	41	< 0.05	$\left({f}_{367}\right)$

For each missing value, KNNImputer finds the *k* other non-missing values that are most similar to the missing value by evaluating the corresponding distance measurements. The missing values are then replaced with a weighted average of the k closest non-missing values, with the weights defined by their similarity distances from the missing value, which in this study was calculated based on the Euclidean distance method. The most challenging part of utilizing KNNImputer is determining the value of k and selecting the neighbors. To address the challenges associated with defining the value of k, the value of k is derived using only the values of non-missing cells (Additional file 3 and Additional file 4).

### Feature selection

The action diagnosis (AD) variable in the AUD-Dataset is critical, as it determines the patients' admission to the hospital based on the International Classification of Disease 10th edition (ICD-10) [45]. The AD variable contains 850 unique ICD-10 codes, which are reduced to 350 level 3 codes based on the hierarchical structure. With 367 features in the AUD-Dataset (listed in Table 1), the goal was to select the most relevant and highly correlated features with class labels. To achieve this, recursive feature elimination with cross-validation [46] using an RF classifier (RFECV-RF) was adopted from Chen and Meng [47] to select the best subset of features.

RFECV-RF is an embedded feature selection technique based on feature ranking (a filter feature section method) and candidate subset selection (a wrapper feature selection method). The aim of this approach is to address the constraints associated with filter and wrapper methods by employing a combined or hybrid technique. RFECV-RF develops models iteratively by deleting features revealing dependency and collinearity and then builds models using the remaining features until all the AUD-Dataset's features are utilized. In this method, the RF classifier is first trained with the training set, and then the relevance of each feature is determined based on its impact on the classifier performance. Afterwards, features are ranked and stored in descending significance order, and the least important feature is omitted from the list. The remaining features are then utilized to build a new classifier, and the performance of the subset of features for the newly built classifier is measured. This technique is repeated iteratively until the feature subset is empty. There will ultimately be a record of classification performance for each subset of features. The performance of each trained RF classifier is assessed using a fivefold cross-validation technique, and a list is constructed to record the validation score of each potential feature subset. Ultimately, the subset of features with the highest predictive accuracy is chosen as the optimal subset of features.

### Imbalanced class distribution

According to Zhu et al. [48], the AUD-Dataset has an imbalanced class distribution, posing a significant challenge during the premodelling phase. The literature suggests various solutions to address this issue, including approaches at the premodelling and algorithm levels and hybrid approaches [49, 50]. These solutions aim to mitigate the impact of an imbalanced class distribution by balancing the class ratios. The most commonly used methods for handling imbalanced class distributions at the premodelling level are resampling techniques, which can be categorized as oversampling, undersampling, or hybrid sampling [51].

SMOTE (synthetic minority oversampling technique) [52] is an oversampling approach that generates synthetic samples to increase the number of instances in minority classes. However, it may poorly characterize class clusters, as certain majority class samples may infiltrate the minority class space [53]. To address this issue, Batista et al. [53] developed SMOTE-ENN by combining Wilson's edited nearest neighbour (ENN) rule [54] with SMOTE. SMOTE-ENN eliminates noise from the majority class samples and removes noisy samples that occur on the incorrect edge of the decision border before balancing the minority class. This approach enhances the prediction performance, leading to exceptional accuracy.

### Data modelling

Wolpert [55] invented the SE method. Unlike other previous ensemble learning methods, stacking combines many types of ML algorithms using meta-learning. In a stacking structure containing two levels, the meta learner (Level-1) combines the outputs of multiple base learners (Level-0). Figure 1 Modelling shows a schematic representation of the stacking structure used in this study, which consists of three stages: i) the training of the base classifiers denoted by the SVM, KNN, DT, RF, and XGBoost algorithms; ii) collecting the output predictions (feature vectors) of the base classifiers to generate a new reorganized training set; iii) the training of the meta classifier using the linear regression (LR) algorithm with a new training set for the development of AUDPM. Descriptions of the developed ML algorithms are presented in Table 2.

We utilized a fivefold stratified cross-validation grid search to select all classifier hyperparameters. In this approach, all potential parameter values are considered. The models are then trained with four training folds for every combination of these parameters, and the test fold that is not used in training is applied to evaluate the results. Finally, the mean of the findings is considered. The hyperparameters with the greatest mean are selected as the optimal hyperparameters.

**Table 2:** Description of developed machine learning algorithms

Model	Description
SVM [56]	It is a statistical model that performs classification using a maximum margin. SVM classifies data by calculating a hyper plane that separates points in an N-dimensional space (N features), while maintaining a maximum margin between points in the classes. To perform classification, the algorithm looks for the hyper plane that separates classes so that the support vectors are furthest from it
KNN [57]	As a non-parametric classifier, KNN attempts to classify an unknown instance based on its neighbors' classification. This means that it labels targets by checking class labels of the k nearest points in the feature space. When classifying a target, it assigns the most common class assigned to its nearest k neighboring points
DT [58]	It is recursive, greedy algorithm that implements a tree data structure where nodes and branches represent targets and features respectively. The first node is the root node, and other nodes split from it. All nodes and subsequent leaves are used in finding the best class for the target. The DT algorithm first develops a tree to its maximum depth, ensuring so each leaf node is pure, and then prunes upwards to optimize the classification error as well as the proportion of final nodes in the tree
RF [59]	It is a bagging ensemble algorithm that is very popular in health-related studies. In general, a RF is a set of classifiers made up of decision trees created from two separate randomization sources. Firstly, a random sample is trained on each individual decision tree, replacing original information with the same size as the supplied training set. Around 37% of redundant instances are estimated to be present in the resulting bootstrapping
XGBoost [60]	It is a DT ensemble based on gradient boosting algorithm that is adaptable, portable and efficient. XGBoost uses the 2^nd^ order derivative as an approximation and provides additional hyperparameters. As a starting point, a predicted value is assumed. Improvement of the prediction accuracy is done by adding an additional tree to the residuals of its preceding tree. After each tree is trained, its contribution to the final model is weighted by a learning rate
LR [61]	The LR algorithm is a common classification approach in clinical research since the dependent event is discrete, such as positive/negative, and it is often included into the ensemble framework. In our work, LR classifies by calculating the probability of a discrete binary class, such as AUD-Positive/AUD-Negative. LR is a type of linear regression that employs a "Sigmoid Function" cost function. This function converts any value between 0 and 1 to the probability value between 0 and 1. Predictions and probability are correlated using this function. The cost function reflects the purpose of optimization. This optimization is accomplished by reducing the cost function in order to create minimum error. Using the gradient descent, the cost value is reduced
SE [62]	The stacking method is a well-liked heterogeneous ensemble learning technique that uses metamodels to enable merging various base classifiers to generate predictions with a higher degree of accuracy. The main benefit of SE is its ability to combine various effective models to produce more accurate forecasts. Particularly, each of base classifier has its own advantages. SE is basically trained on the entire training sent and a meta estimator is employed to learn how to combine the base classifiers, distinct other ensemble learning algorithms such as RF. SE can evaluate the error of all base classifiers individually using basic learning processes, and then decrease residual errors using meta learning steps

*SVM* Support vector machine, *KNN* K-nearest neighbor, *DT* Decision tree, *RF* Random forest, *XGBoost* Extreme gradient boosting, *LR* Logistic regression, *SE* Stacking ensemble

### Evaluation

The performance assessment of the developed classifiers involved utilizing the receiver operating characteristic (ROC) curve, along with the area under the ROC curve (AUROC), the area under the precision-recall curve (AUPRC), precision, recall, F1-score, and overall prediction accuracy (ACC). The evaluation process encompassed the determination of these performance metrics by referencing values for true positives (TP), false positives (FP), false negatives (FN), and true negatives (TN). Particularly in datasets with imbalanced distributions, like the AUD-Dataset, the AUPRC holds greater informative value compared to the AUROC [63]. The AUPRC is alternatively known as the average positive predictive value or average precision [64]. Descriptions of the evaluation metrics are presented in Table 3.

**Table 3:** Description of performance metrics and their formula

Metric	Description	Formula
Precision	Precision or Positive Predictive Value (PPV) is a performance metric that determines how many of the records that were expected to be positive were truly positive. The main aim of looking at this number is to decrease the number of false positives	$Precision= \frac{TP}{TP+FP}$
Recall	Recall or True Positive Rate (TPR) describes the sensitivity of the classifier. The number of positive samples captured by accurate forecasts is measured by Recall. When all positive samples must be identified, and all false negatives must be avoided, Recall is considered as a performance metric	$Recall (Sensitivity)= \frac{TP}{TP+FN}$
F1-Score	The F1-Score is calculated by averaging Precision and Recall. As a result, it shows the performance of the classifier in detecting positive records. This means that the classifier performs best in the positive class if the F1-Score is high. For binary classifications based on imbalanced datasets, F1-Score can be a more appropriate metric to be considered than accuracy	$F1-Score=2\times \frac{Precision \times Recall}{Precision+Recall}$
Predictive Accuracy	The most popular measure of the classifier’s performance is predictive accuracy, which evaluates the algorithm's overall effectiveness by calculating the likelihood of the class label's actual value. Measuring the predictive accuracy is the fastest way to understand whether the predictive model has been trained correctly and what the overall performance is. However, it is not the best option to be considered since it cannot give detailed information about the performance of the classifier	$Accuracy= \frac{TP+TN}{TP+TN+FP+FN}$
AUROC	The AUROC is a single number that measures the total area underneath the ROC curve and thereby summarizes the performance of the classifiers, as long as we assume that FP and FN are equal mistakes. In most medical situations, FN is considered more serious as these people are not identified by the test. Individuals given an FP classification will be tested further, which provides the opportunity to change the classification. ROC curve visualizes the trade-off between TPR and False Positive Rate (FPR) by displaying them for various threshold settings (cutoff points). In particular, the ROC curve attempts to map the cumulative distribution function of a defined probability distribution in the y-axis against the x-axis, for both true and false identified events. In this curve, the y-axis is the TPR, and the x-axis is the FP rate which is calculated as	$False\, Positive \,Rate= \frac{FP}{TN+FP}$
AUPRC	The AUPRC is another widely used performance metric in binary classification problem. It is a threshold-independent measure that estimates the area under a curve formed by a trade-off between several characteristics of performance as the model's prediction threshold changes. In the AUPRC curve, Recall is on the x-axis and Precision is on the y-axis	

*AUROC* Area under receiver operating characteristics curve, *AUPRC* Area under the precision-recall curve

Due to the imbalanced distribution of classes, a stratified split of 80% for the training set and 20% for the test set was adopted. Additionally, within the training set, 20% of the data were set aside for model validation. The ratio of AUD-Positive and AUD-Negative records is constant in the training and testing sets when using a stratified split approach. All preprocessing techniques, including missing value imputation, feature selection, imbalanced class distribution, scaling, etc., are only learned from the training set and then applied to the test set with the necessary modifications. In this way, data leaks from the test set to the learning process are avoided, which might result in an overly positive assessment of the model performance. This indicates that the test set did not contribute to the learning process and was solely used to evaluate the performance of the final models.

### Application for the early detection of patients with AUD

**Fig. 3:**
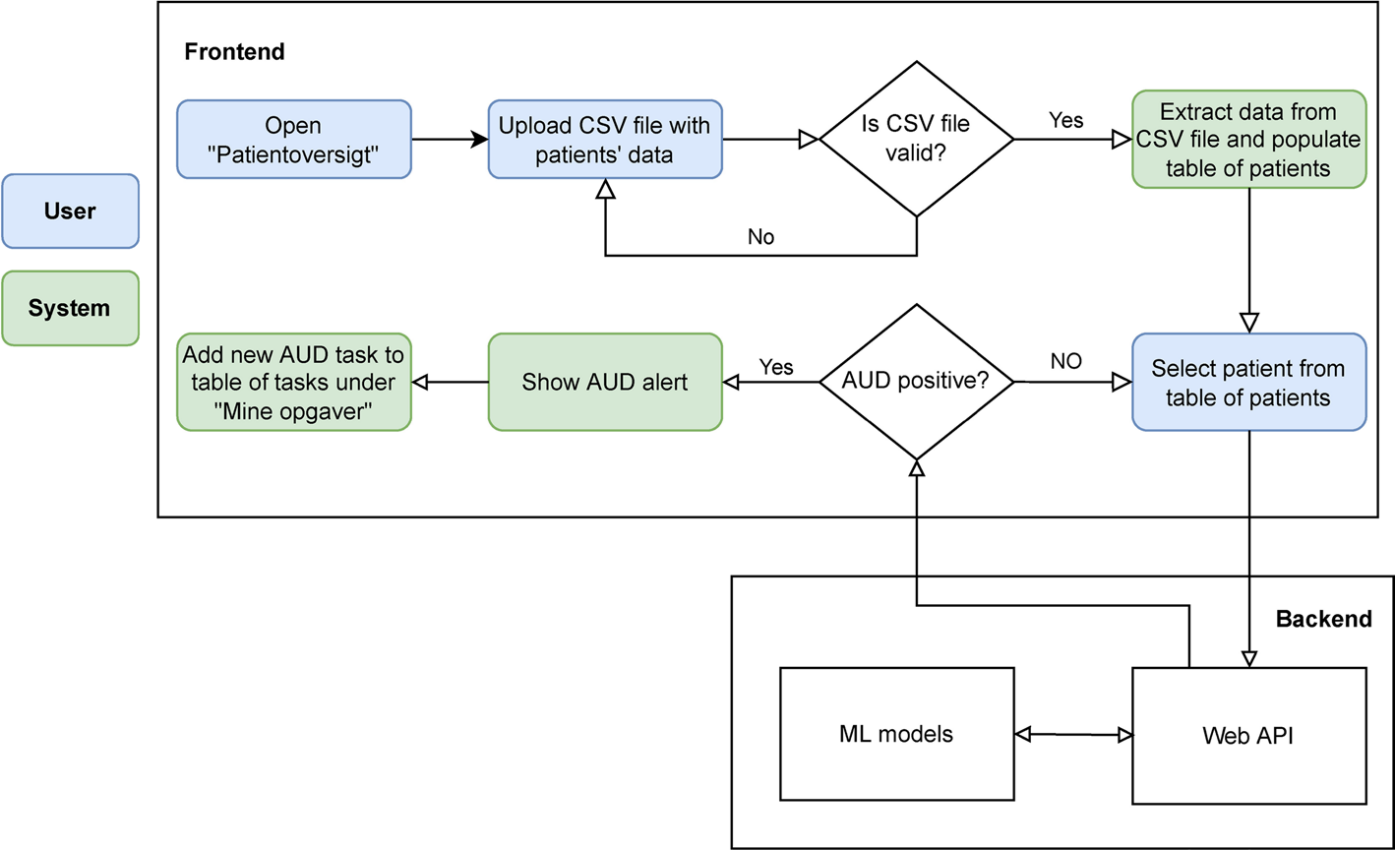
Workflow of the AUD-DSS prototype

To make the best performing prediction model operational, a web-based prototype AUD decision support system (AUD-DSS) was developed. The development process followed a codesign approach involving medical staff as the relevant stakeholders in all steps of the process. Early in the process, the current workflow of medical staff was analysed to enable AUD-DSS to be best fit into and support the current workflow and provide a simple and convenient way for medical staff to diagnose patients as a natural part of the already existing workflow. This was an explicit wish of the medical staff. AUD-DSS was developed and evaluated through a few codesign iterations. The workflow of the AUD-DSS prototype is presented in Fig. 3.

### Ethical approval

The collection of data for the Relay Study was approved by the Danish Data Protection Agency (The Region of Southern Denmark project-ID 2008-58-0035). The Review Board at the Regional Scientific Ethical Committees of Southern Denmark decided that formal informed consent was not required of the patients, as the study was considered a register study that did not entail intervention (Project ID: S-20130084). The collection of data from electronic health records was approved by the Danish Patient Safety Authority (Project-ID 3-3013-1601/1) and the Danish Data Protection Agency (The Region of Southern Denmark Project-ID 16/12126).

## Results

### Data preparation

As mentioned in Sect. "AUD dataset", we collected a multidimensional dataset from 2551 patients to train ML algorithms for the detection of patients with AUD. Of the 367 features in the AUD-Dataset, six features had missing values, which mostly appeared in the class of AUD-Positive patients. Body temperature, with 50% missing values in the AUD-Positive class and 8% missing values in the AUD-Negative class, had the highest missing values among all features. On the other hand, diastolic blood pressure and systolic blood pressure had the fewest missing values. Moreover, there were no missing values in the outcome variables (AUD-Negative and AUD-Positive), and they were used as informative variables to impute the missing values. As mentioned in Sect. "Missing value imputation", KNN-Imputer was used to impute missing values in the AUD-Dataset.

**Fig. 4:**
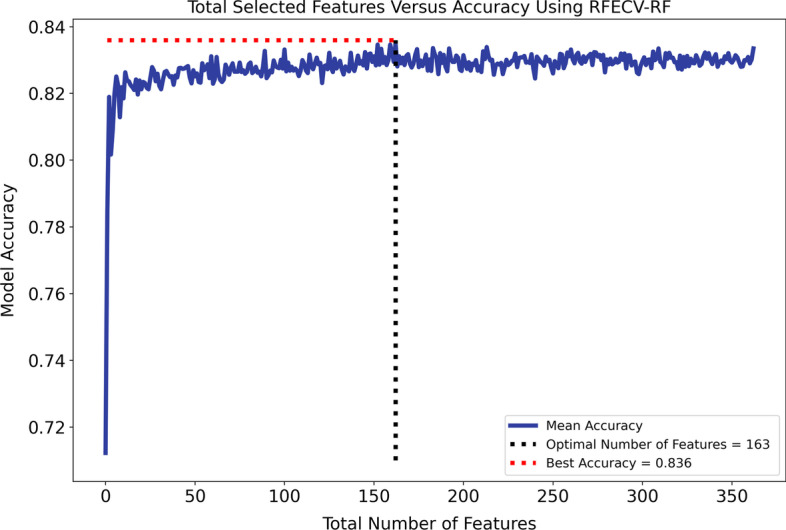
Feature selection impact on number of features

**Fig. 5:**
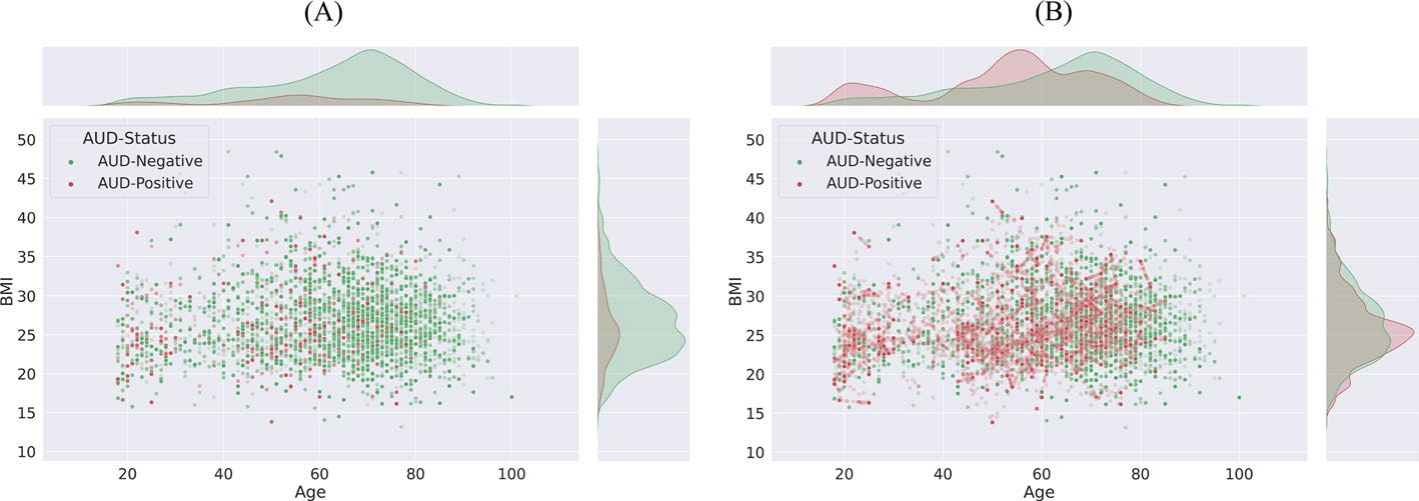
Data distribution of attributes Age and BMI before (**A**) and after (**B**) SMOTE-ENN implementation

As discussed in Sect. "Feature selection", one of the main challenges in analysing the AUD-Dataset is its high dimensionality. To overcome this problem, we used RFECV-RF based on fivefold cross validation. As presented in Fig. 4, RFECV-RF reached the best accuracy of 86% in iteration 163, thereby reducing the number of features from 367 to 163. As described in Sect. "Imbalanced class distribution", we used SMOTE-ENN to balance the AUD-Dataset. Figure 5a, b show the distributions of the imbalanced and balanced AUD-Dataset based on two features, including age and BMI. As shown in these figures, the number of records in the AUD-Positive class increased after applying SMOTE-ENN. Furthermore, the number of records before and after data balancing is presented in Table 4.

**Table 4:** Number of records in imbalanced and balanced datasets

Class	Imbalanced	Balanced
AUD-Negative	7964	7596
AUD-Positive	1611	6486

### Model evaluation

In this section, the results of models developed based on the proposed methods are compared with baseline models developed before applying the proposed method. This means that the baseline models are developed based on the AUD-Dataset, which is not preprocessed by any of the imputation, feature selection, or sampling techniques.

The optimal hyperparameter values for each ML algorithm are listed in Table 5. The average performance of the different trained ML algorithms with the test set for the baseline and proposed method is presented in Table 6. The DT model from the baseline group had the lowest performance among all classifiers. On the other hand, based on our proposed method, the SE model achieved the highest recall, F1-score, and AUPRC, RF achieved the highest precision, and both models achieved the same accuracy. Although RF, in terms of precision, outperformed the other models by 0.98, SE and SVM each achieved a comparable precision rate of 0.96 (Table 6). From Fig. 7 and Table 6, it can be concluded that SE, based on our proposed method, is the best performing model for the early detection of patients with AUD. Therefore, we refer to this model as AUDPM, and we dive deeper into the results achieved by this model based on the test set. As shown in Table 6, AUDPM outperforms and is comparable with other predictive models in terms of precision, recall, F1-score, and accuracy, with values of 0.96, 0.94, and 0.95, respectively. In terms of precision, AUDPM is comparable with RF and SVM, with values of 0.98 and 0.96, respectively.

**Table 5:** Configurations of classifiers

Model	Hyper-Parameters
Random forest	Number of trees in the forest = 50, maximum depth of each tree = 20, the minimum number of samples to split each node = 8
XGBoost	Learning rate = 0.3, maximum depth of each tree = 6, minimum loss reduction to split each node = 1, regularization term on weights = 20, subsample ratio of columns for each tree = 0.5
Decision tree	Maximum depth = 12
K-nearest neighbor	Number of k = 7
Support vector machine	Kernel = Radius basis function, C = 1, Gamma (γ) = 0.001
Logistic regression	Batch size = 100, Debug = True, Standardize attribute = True, Maximum number of iterations to perform = 100, Ridge value in the likelihood = 1.0E-8, conjugate gradient descent = True

Accuracy is commonly regarded as among the most significant metric for evaluating ML algorithms. As stated previously, six classifiers were utilized to evaluate the effectiveness of the proposed method. As shown in Table 6, the accuracies of the baseline models are 0.89, 0.90, 0.85, 0.88, 0.87, and 0.88 for the AUDPM, RF, DT, KNN, SVM, and XGBoost classifiers, respectively, with 367 features and an imbalanced class distribution in the AUD-Dataset. In considering the accuracies of all classifiers based on the proposed method, AUDPM and RF each achieved an excellent accuracy of 0.97. The results of other classifiers also showed a great improvement in the accuracy, with accuracy values of 0.92, 0.88, 0.93, and 0.91 for the DT, KNN, SVM, and XGBoost classifiers, respectively.

**Table 6:** Average performance of the developed models based on test set

Model	Precision (positive predictive value)	Recall (sensitivity)	F1-score	Accuracy	AUROC	AUPRC
*Baseline*						
Stacking ensemble (AUDPM)	0.91	0.78	0.83	0.92	0.95	0.70
Random forest	0.94	0.70	0.75	0.90	0.95	0.56
Decision tree	0.74	0.72	0.73	0.85	0.76	0.54
K-nearest neighbour	0.78	0.76	0.77	0.88	0.84	0.61
Support vector machine	0.90	0.62	0.66	0.87	0.86	0.38
XGBoost	0.87	0.73	0.77	0.90	0.73	0.56
*Proposed pipeline*						
Stacking ensemble (AUDPM)	0.97	0.96	0.97	0.98	0.99	0.90
Random forest	0.97	0.89	0.93	0.96	0.99	0.87
Decision tree	0.87	0.80	0.83	0.91	0.91	0.70
K-nearest neighbour	0.79	0.74	0.76	0.88	0.86	0.59
Support vector Machine	0.96	0.81	0.86	0.93	0.95	0.75
XGBoost	0.90	0.73	0.78	0.90	0.73	0.62

As noted previously, recall or TPR is a critical performance evaluation metric that indicates a classifier's sensitivity [65]. Recall is crucial since it demonstrates that AUD-Positive patients are appropriately identified. As displayed in Table 6, the baseline classifiers achieved poor recall ratings. A very poor recall score (slightly exceeding 0.62) was achieved with the SVM classifier, while our proposed method achieved a recall score of 0.81, a significant improvement. AUDPM achieved the highest recall score (0.94) when applied to the 136 features and balanced training set from the AUD-Dataset. RF achieved a recall score of 0.90, which was the second best among all developed classifiers.

**Fig. 6:**
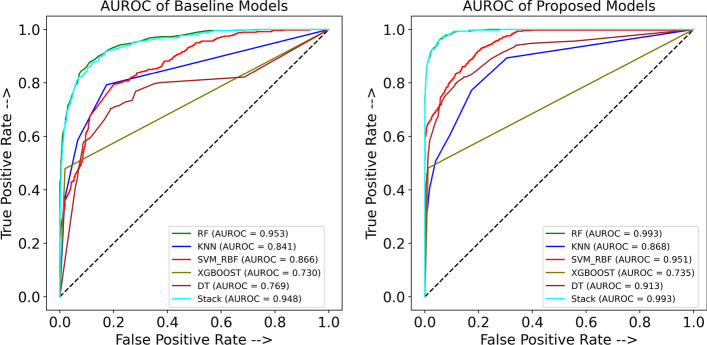
Baseline and proposed method’s result of Area Under the Receiver Operating Characteristic Curve (AUROC) of models including Random Forest (RF), Support Vector Machines (SVM), k-Nearest Neighbors (KNN), Decision Tree (DT), XGBoost, Stacking Ensemble (Stack)

Figure 6 shows the AUROC curves for each classifier trained with the result of the proposed method and the baseline models and reports the AUROC in each case. In ML tasks, ROC curves are utilized to validate the performance of predictive models by indicating the FP rate versus the TP rate [66]. By assessing the intrinsic validity of a test based on the trade-off between the TP (sensitivity) and FP (1-specificity) rates at different cut points on the X and Y axes, respectively [67], the ROC plays a crucial role in binary diagnostic tests (positive and negative tests) [68]. The AUROC provides an effective metric that depicts the area under the ROC curve and is a means to describe the performance quality of a diagnostic model. An AUROC of 0.9 to 1.0 is considered outstanding in the literature [69]. Moreover, the better AUROC in the proposed models in comparison to the baseline models is an important factor that shows that the preprocessing and feature section methods could improve the performance of classifiers [70]. As seen in Fig. 6 (Baseline), the AUROC for the XGBoost classifier trained by baseline features and imbalanced class was 0.71, which is the worst result, followed by DT, with an AUC of 0.75. The performance of both classifiers improved after considering our proposed method, with AUCs of 0.93 and 0.76 for DT and XGBoost, respectively. On the other hand, based on our proposed method, the AUDPM and RF classifiers each achieved an AUC value of 0.99 (Fig. 6), the best AUC value among all trained models.

**Fig. 7:**
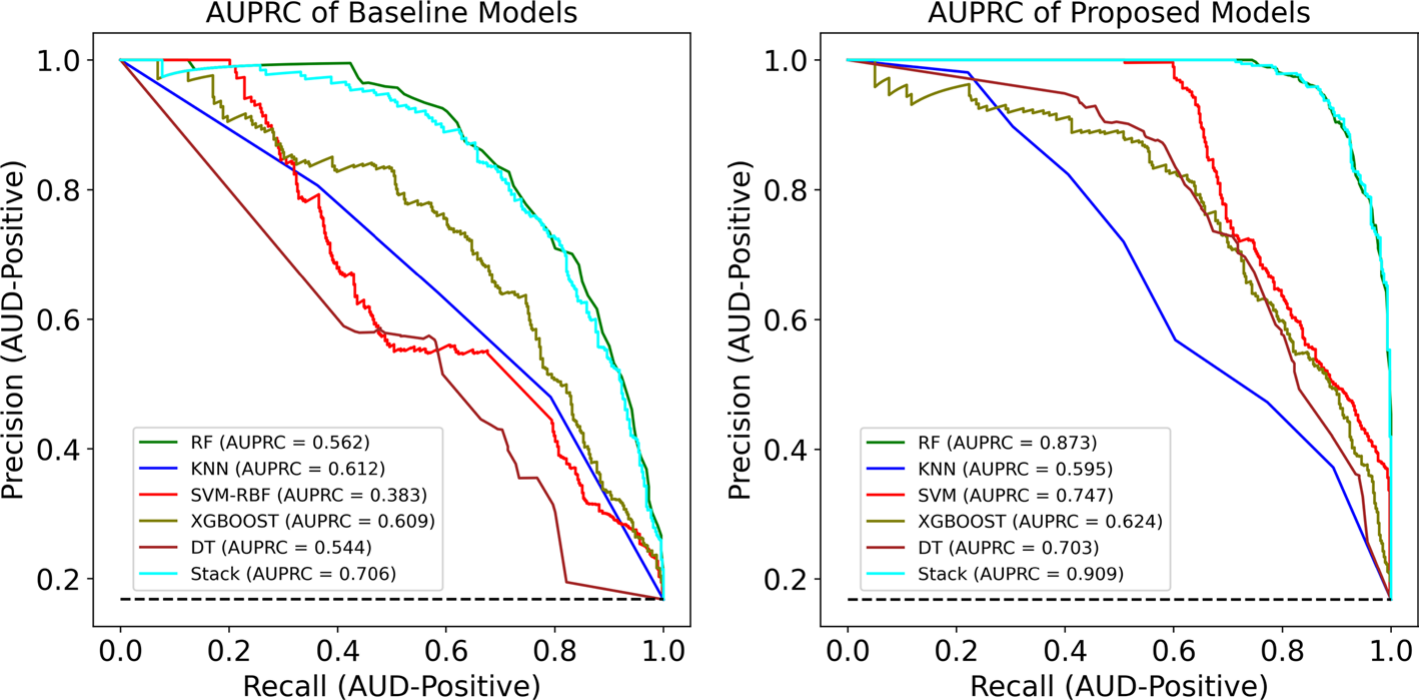
Baseline and proposed method’s result of Area Under the Precision-Recall Curve (AUPRC) of models including Random Forest (RF), Support Vector Machines (SVM), k-Nearest Neighbors (KNN), Decision Tree (DT), XGBoost, Stacking Ensemble (Stack)

Figure 7 shows the AUPRC for each classifier trained based on our proposed method as well as the baseline models. It should be noted that the baseline of the AUPRC (the black dotted line in each curve) is equal to the AUD-Positive fraction. Since the AUD-Dataset consists of approximately 17% AUD-Positive and approximately 83% AUD-Negative examples, the baseline AUPRC is 0.17. As shown in Fig. 7, the best model is AUDPM trained based on our proposed method. In considering the 163 features selected by RFECV-RF and the balanced training set obtained using SMOTE-ENN, a notable result of over 0.92 was obtained for the AUPRC with the SE algorithm. This is the highest among all developed models. When SE was applied to the 367 features and the imbalanced training set, it achieved an AUPRC of approximately 0.55. This is the second-worst performance among all baseline models. RF also obtained a good AUPRC score of 0.89 based on our proposed method. The precision-recall curve, which illustrates the recall versus precision for all feasible thresholds, is one of the most popular and recent predictive performance measures for evaluating the effectiveness of classifiers in the area of medical research. It has been implied through multiple studies that the AUPRC is more insightful than the ROC curve and AUROC for assessing a risk model's prediction performance with an imbalanced class distribution [71], such as in this study, where the distribution of samples in the AUD-Positive class is low. The AUPRC does not include the number of TN since it is the area under the curve of the plot of recall versus precision across thresholds, and precision is dependent on the records that were assumed to be AUD-Positive and were truly AUD-Positive.

### Clinical factor identification

**Fig. 8:**
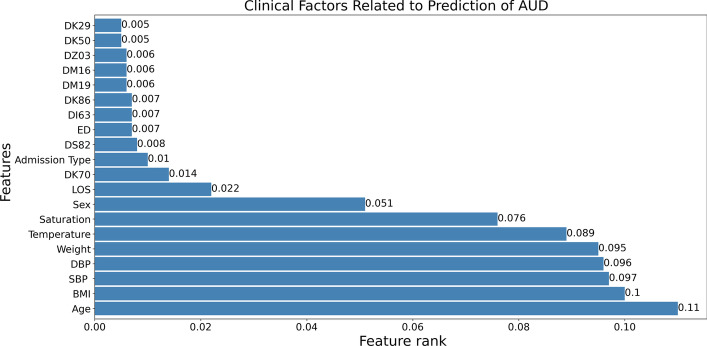
Top 20 important features extracted by RFECV-RF, ranked by Gini Index. BMI (Body mass index), SBP (Systolic Blood pressure), DBP(Diastolic Blood pressure), DK70 (Alcoholic liver disease), DS82(Fracture of lower leg, including ankle), DI63 (Cerebral infarction), DK86 (Other diseases of pancreas), DM19 (Another arthrosis), DM16 (Osteoarthritis of hip), DZ03 (Encounter for medical observation for suspected diseases and conditions ruled out), DK50 (Crohn's disease), DK29 (Gastritis and duodenitis)

As seen in Fig. 4, RFECV-RF significantly reduced the number of features from 367 to 163, and it could likewise improve the classification performance. The features selected by RFECV-RF are ranked based on their significance, and the top 20 are presented in Fig. 8. The features are ranked based on their Gini index (GI) [72], in which higher values indicate more important features. It was found that age, BMI, systolic and diastolic blood pressure, and weight are among the most important factors for the early detection of individuals with AUD.

Systolic and diastolic blood pressure are the other features listed among the top five most important features related to the early detection of patients with AUD; see Fig. 8. These two features have also been mentioned in many previous studies (on drinking problems) as being highly correlated with heavy drinking and increased blood pressure [73, 74]. In terms of ADs, DK70 (alcohol-induced liver disease), DS82 (fracture of the lower leg, including the ankle), DI63 (cerebral infarction), DK86 (other diseases of the pancreas), and DM19 (another arthrosis) are the top five AD factors. Clinical factors and comorbidities associated with the prediction of AUD have been identified in our previous studies [34, 75], which provide a more in-depth analysis of ADs. However, no study has examined clinical and risk factors such as systolic and diastolic blood pressure, BMI, weight, saturation, temperature, and AD for the prediction of patients with AUD from EHRs in a single study.

### AUD-DSS prototype

To use the AUD-DSS prototype, the medical staff must import patient data by uploading a comma-separated values (CSV) file containing the data. In the final version, these data will be loaded automatically from EHRs (EPJ Syd). Figure 3 shows the user interface for the EHR listing of the names of the patients who the doctor needs to examine next (the names on the list are made up for the purpose of this user interface walkthrough).

**Fig. 9:**
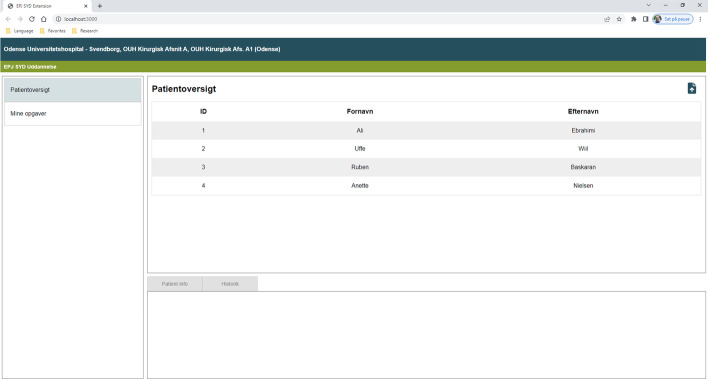
AUD-DSS user interface

**Fig. 10:**
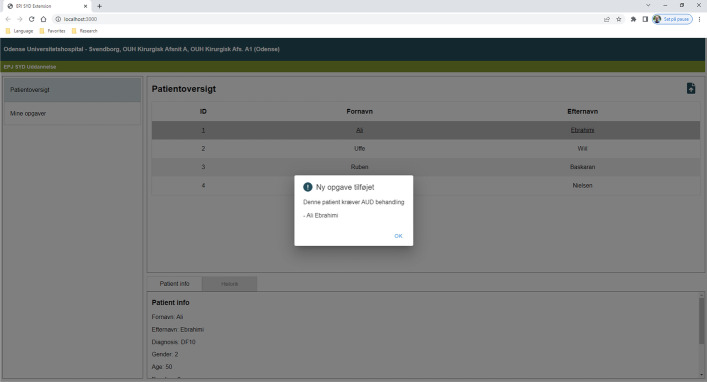
The AUD Positive alert adding a new task to the task list (Mine opagver). Ny opgave tilføljot (New task added), Denne patient kræver AUD behandling (This patient requires AUD treatment)

When the medical staff clicks on a patient’s name, that patient's data are sent to the AUD-DSS backend implementing AUDPM. After the backend has executed AUDPM, it returns a result of either “AUD-Positive” or “AUD-Negative” to the frontend. If the result is “AUD-Positive”, a pop-up alert is shown to the medical staff (see Fig. 9), and a new task is added under the My Tasks page ("Mine opgaver" in Fig. 9). The My Tasks page contains a list of tasks associated with the selected patient during consultation. In this way, the medical staff is alerted in case AUD-DSS detects a patient who may suffer from AUD, and they can take the appropriate action while examining the patient (see Fig. 10).

Hence, the developed decision support system has been smoothly integrated into the current workflow of the medical staff. Only when AUD-DSS identifies a patient who may be suffering from AUD does the system take action and add a task to the task list as mentioned above. This complies with the wishes of the medical staff involved in the codesign process–to best fit into and support the current workflow with the addition of the new functionality. The user interface in the AUD-DSS prototype is similar to the one in the EHR to best support a future integration of AUD-DSS into EHRs.

In summary, the developed AUD-DSS is intended to help medical staff with the early detection of patients with AUD and improve clinical decision-making effectively and efficiently. Therefore, intervention can be conducted earlier to prevent more complications caused by late AUD diagnosis.

## Discussion

We proposed an effective prediction model for the early detection of patients with AUD. Our proposed AUDPM was designed by integrating KNN-Imputer, RFECV-RF, SMOTE-EEN, and SE with two levels. KNN-Imputer was applied to predict missing values, RFECV-RF was applied to reduce dimensionality and select the best subset of features, SMOTE-EEN was used to remove noise and balance the training set of the AUD-Dataset, five ML algorithms, including SVM, KNN, DT, RF, and XGBoost, were considered as base learners, and LR was utilized as the meta-learner to learn and generate AUDPM. A multidimensional dataset from two sources was utilized through a combination of a questionnaire and EHRs of patients from the Region of Southern Denmark.

We evaluated the performance of algorithms before and after applying our proposed method to existing challenges by examining various metrics. Each metric focuses on a special aspect of the performance. Except for the AUROC and AUPRC, all metrics were constructed based on a confusion matrix (TP, FP, TN, and FN). The performance of all developed algorithms improved after applying the proposed method. However, AUDPM achieved the highest overall performance. Our results showed that applying the proposed method could improve precision, recall, and F1-score in all investigated algorithms except KNN, whose performance declined in terms of recall and F1-score. Although utilizing RFECV-RF, a hybrid technique, is a computationally expensive technique, it addresses the limitations of filter and wrapper feature selection techniques [76]. This is also the case for SMOTE-ENN, which is a hybrid balancing technique that address the lack of a noise reduction step in our proposed method. Furthermore, we designed and developed the proposed AUDPM into the AUD-DSS for the early detection of patients with AUD effectively and efficiently. AUD-DSS gathers patient EHRs and personal information and transmits them to a secure web server through an API. The proposed AUDPM is then loaded to detect the patients’ current AUD status and return a result to the frontend of either AUD-Positive or AUD-Negative.

### Clinical factors

Clinical factors related to the prediction of patients with AUD were also presented. In the literature, risk factors such as gender and age have been discovered in many studies [27, 30, 34]. In comparison to the work in [34], we can see that age was still the most important factor related to the early detection of patients with AUD. As shown in Fig. 8, BMI and weight were found among the top five factors (the second and fifth most important factors, respectively), which were also discovered in many previous studies related to drinking problems. In recent years, alcohol usage has likely contributed to the extra energy intake linked with BMI increases in certain individuals [77]. Age, gender, and the frequency and amount of alcohol consumed are highly related to the increases in BMI and weight. The correlation between alcohol consumption and BMI is often higher in males than in females [78], particularly due to the quantity and kind of alcohol consumed by men. Furthermore, many previous studies [77] have found that heavy drinkers have a higher BMI and weight than nondrinkers. This can explain why these two factors are among the top five most important features in this study.

### Comparison to previous study results

Different studies have evaluated the application of ML techniques for the early detection of AUD. Afzali et al. [24] compared six algorithms, including SVM, RF, ANN, ridge-net, elastic-net, and LASSO regression, using two datasets, a Canadian sample of 3826 secondary school students and an Australian sample of 2190 secondary school students. Their method only consisted of a multivariate imputation technique, which resulted in the best performance on elastic-net with an accuracy of 0.87 and an AUROC of 0.70. The main limitation of their research was that they did not consider the feature selection and balancing steps, which are important steps in the development of a predictive model. Furthermore, even though their data were collected from two different locations (Canada and Australia), all the collected data were based on self-reported responses to questionnaires. In the current study, we addressed the feature redundancy and imbalanced class distribution problems, and we also collected a multidimensional dataset from different sources, including the EHRs of patients as well as self-reported responses to questionnaires.

In another study, Silveira et al. [29] compared the results of five ML algorithms, including SVM, LR, AdaBoost, gradient boosting, and RF, with a dataset consisting of questionnaires and MRIs collected from 392 youth. They applied SMOTE to balance the dataset, and they achieved an accuracy of 0.80 with the RF model. However, they did not consider noise removal and feature selection methods. Bonnell et al. [25] analysed the data of 43,545 adults collected through questionnaires and EHRs. They employed six different ML algorithms, including LR, SVM, KNN, ANN, DT, and RF, and RF achieved the best accuracy of 0.76 and AUROC of 0.78 based on the selected feature using information gain filter feature selection. One of the main disadvantages of their study was that they used a filter feature selection method. In such a method, classifier biases cannot be included in the classification model [34, 79]. Another disadvantage of their study is that noise removal and balancing techniques were not considered.

In summary, the primary contributions of this paper are fourfold: (1) developing AUDPM for the early detection of patients with AUD, which consists of imputation, feature selection, and balancing techniques to address challenges in collected datasets, and feeding to an SE algorithm, (2) analysing and comparing of the performance of the chosen AUDPM with that of state-of-the-art predictive models, where AUDPM demonstrated superior performance; (3) relating the study points to clinical factors that are highly correlated to the development of predictive models to identify patients with AUD; and (4) developing a real-world clinical decision support system for the early detection of patients with AUD. In summary, it is anticipated that the proposed AUDPM and AUD-DSS can aid clinicians in identifying patients with AUD and improve clinical decision-making.

### Limitations and future work

The fact that the data used in developing the predictive models for this study originated from patients who were hospitalized in the OUH gastroenterology, neurology, and orthopaedic departments is one limitation. This may create a risk of bias, which is reflected in the extracted clinical factors. The relatively small patient populations and lack of regional variety that arise from single-site research are additional limitations of our study. We intend to overcome these limitations by carrying out a nationwide Danish study with a dataset including patients from all regions and hospitals to validate the prediction accuracy. Furthermore, the effectiveness of AUD-DSS will be tested in a real-life setting in Danish hospitals.

Moreover, we will consider other imputation and feature selection techniques and data sampling methods. In this study, we considered SMOTE-ENN to remove noise in addition to balancing the AUD-Dataset. In future work, we will also conduct an experiment comparing different outlier detection methods. Although we could use the power of RFECV-RF to extract clinical factors related to the prediction of patients with AUD in this study, in future work, we will consider the explainability and interpretability of the developed models using methods such as Shapley additive explanations (SHAP) [80] and local interpretable model-agnostic explanations (LIME) [81].

## Conclusion

To conclude, we proposed a method to address challenges such as missing values, feature redundancy, noise reduction, and class imbalance in a dataset containing EHRs of AUD-Positive and AUD-Negative patients. We also proposed an SE model combining six different ML models for the early detection of patients with AUD. Our proposed method increased the prediction performance of the developed algorithms in comparison to that of the same algorithms before applying the proposed method. Using hybrid methods in conjunction with stacking approaches resulted in significant improvements in the prediction performance. Our study results could assist researchers in choosing the best way to address challenges in predicting steps and developing predictive models based on EHRs. Furthermore, the newly developed AUD-DSS may assist clinical staff in the early detection of patients with AUD.

### Supplementary Information

**Additional file 1**. TRIPOD checklist.

**Additional file 2**. Figures indicating the distribution of features among AUD status and sex.

**Additional file 3**. Table that displays a list of features used in this study, indicating whether they were considered in the final model and selected during the feature selection process.

**Additional file 4**. Average performance of the developed models based on test set and validation set.

## Data Availability

The dataset used for this study is not publicly available due to the possibility of compromising individual privacy but is available from the corresponding author on reasonable request.
