# Supplementary figures and images for: AUD-DSS: a decision support system for early detection of patients with alcohol use disorder
Source: BMC Bioinformatics. 2023 Sep 2;24:329. doi: 10.1186/s12859-023-05450-6 (PMC10474761; doi:10.1186/s12859-023-05450-6)

**Bellow figures indicated the distribution of feature among AUD statues and sex.**

| 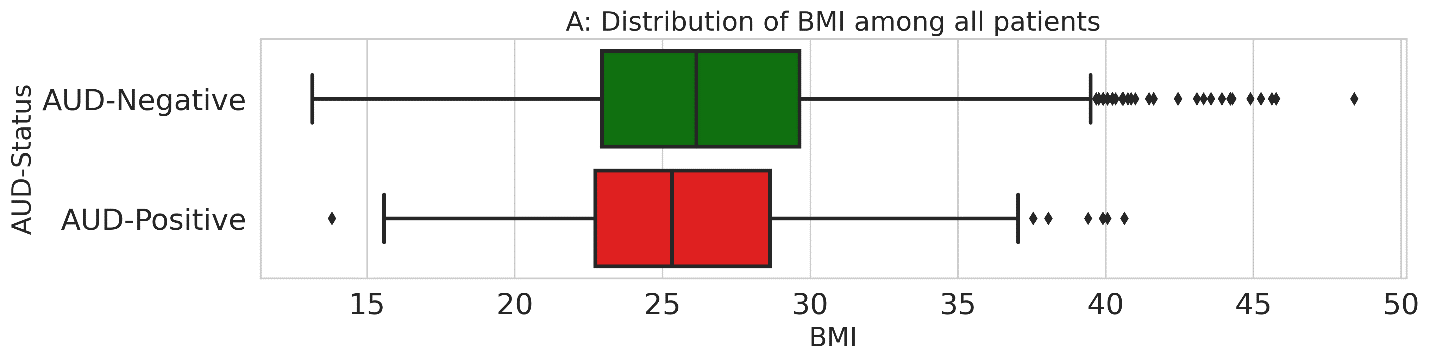 |
| --- |
| 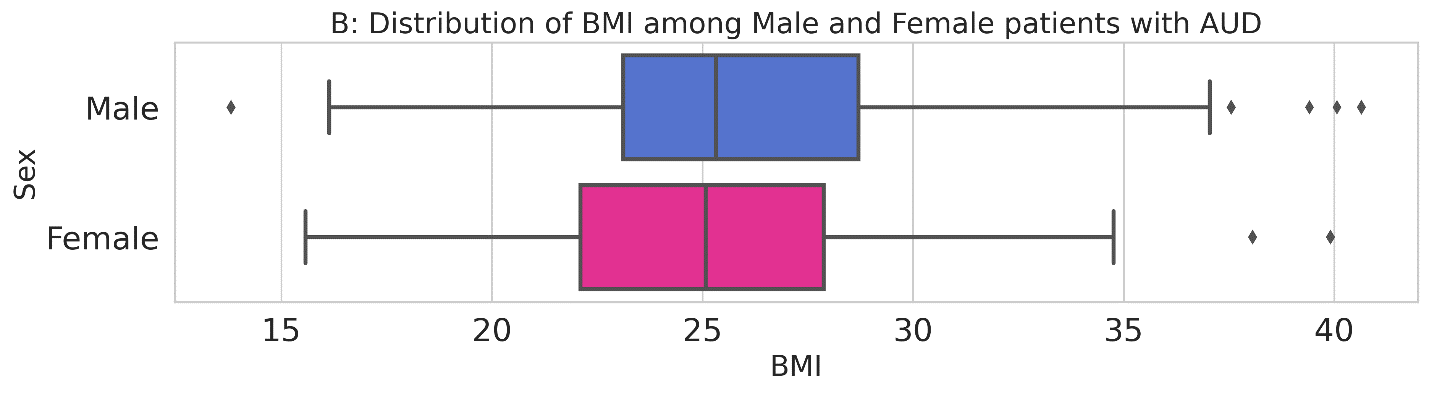 |
| 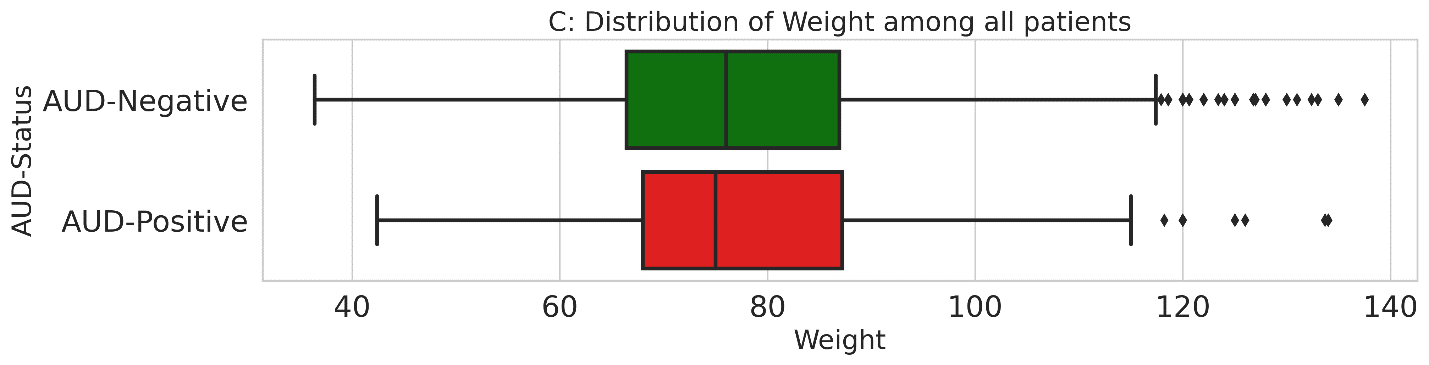 |
| 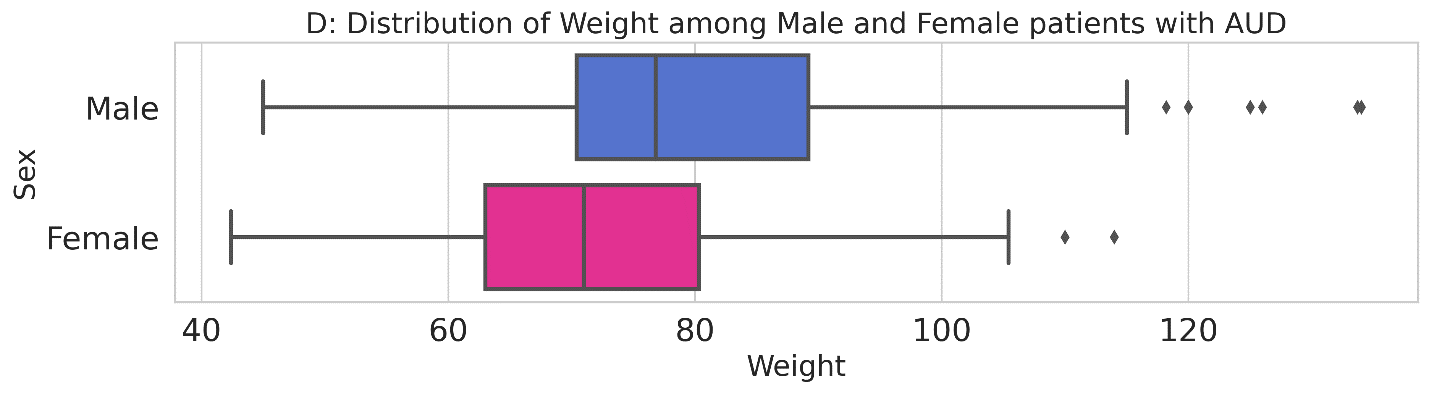 |
| 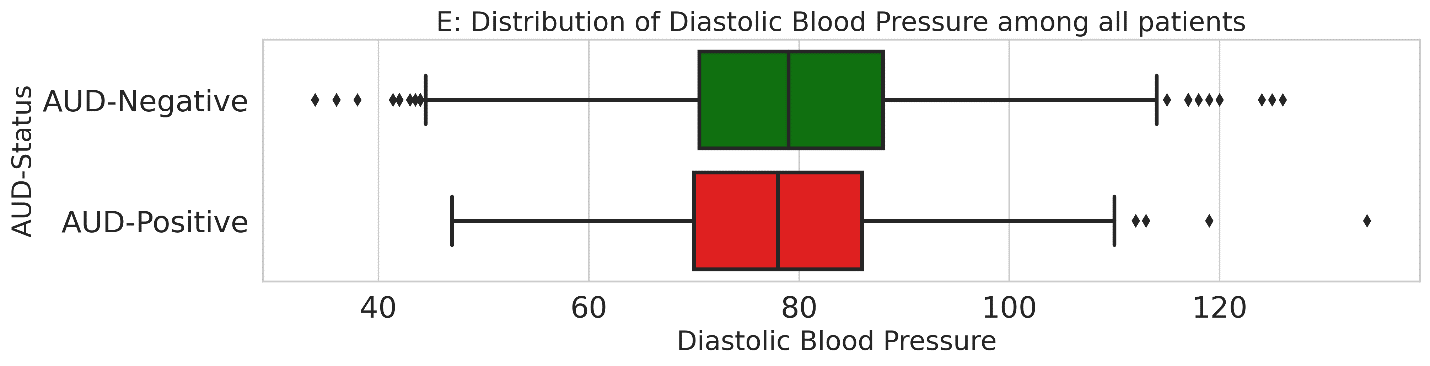 |
| 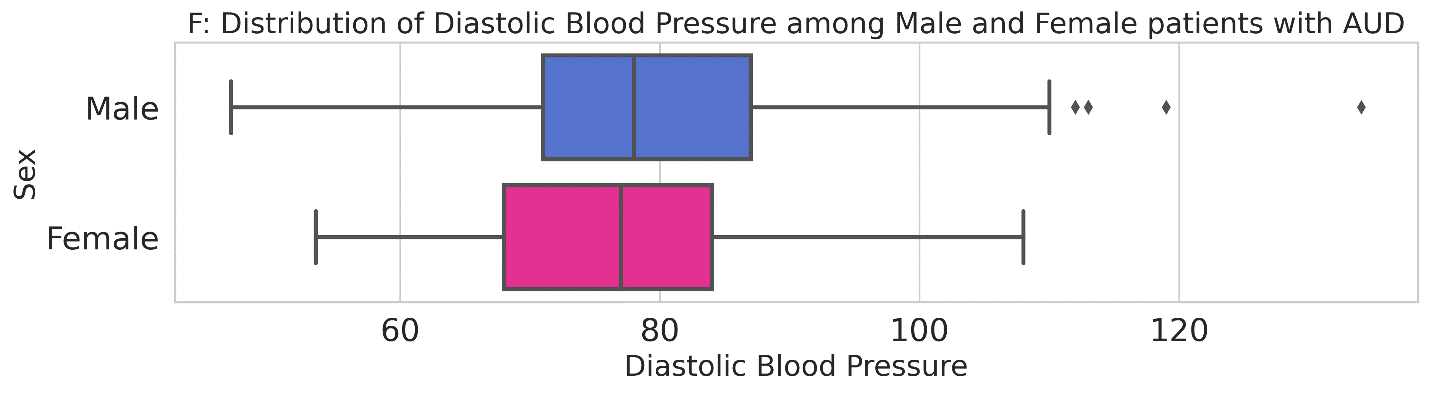 |
| 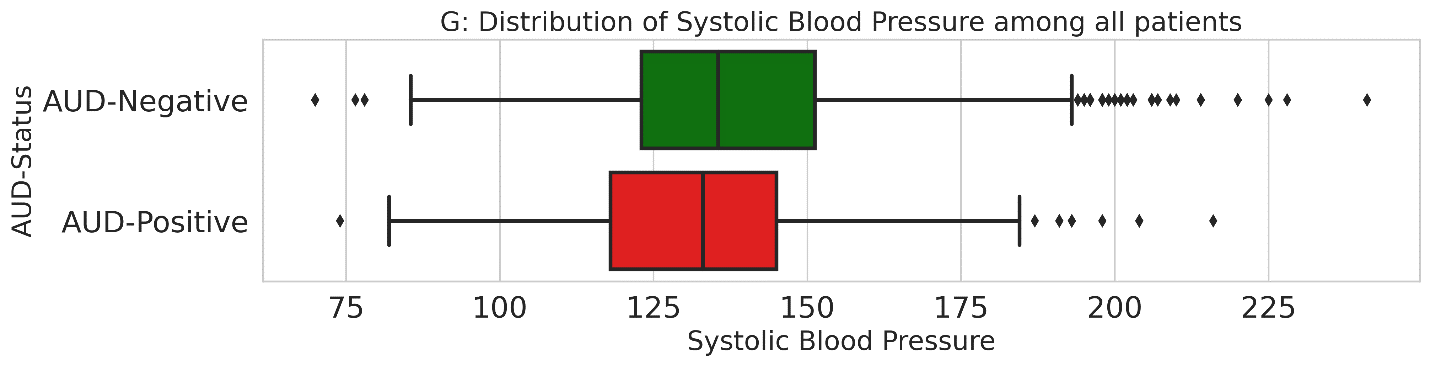 |
| 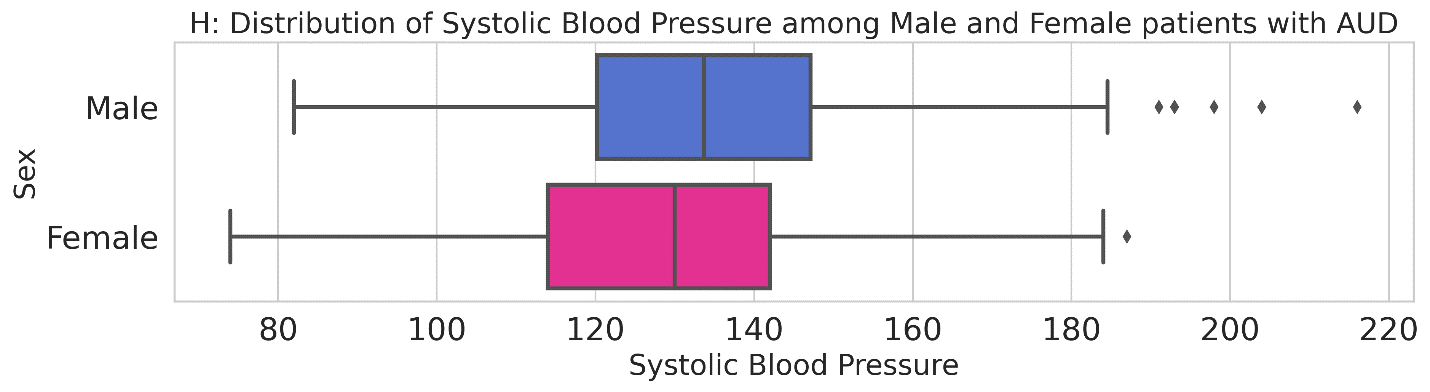 |

Supplement: Supplementary file 2 — Additional file 2. Figures indicating the distribution of features among AUD status and sex. [file 12859_2023_5450_MOESM2_ESM.docx]
