# Supplementary material for: AUD-DSS: a decision support system for early detection of patients with alcohol use disorder
Source: BMC Bioinformatics. 2023 Sep 2;24:329. doi: 10.1186/s12859-023-05450-6 (PMC10474761; doi:10.1186/s12859-023-05450-6)
Supplement: Supplementary file 3 — Additional file 3. Table that displays a list of features used in this study, indicating whether they were considered in the final model and selected during the feature selection process. [file 12859_2023_5450_MOESM3_ESM.docx]

Table 1 displays a list of features used in this study, indicating whether they were considered in the final model and selected during the feature selection process.

| **Table 1. Full list of features.** | | |
| --- | --- | --- |
| **Variable** | **Description** | **Included in final model** |
| Gender | Male or Female | Yes |
| Age | Age of patient at time of Relay study. | Yes |
| Admission_type | Admitted patients or outpatients. | Yes |
| LOS | The amount of time the patient spent at the hospital for each visit. | Yes |
| ED | If the patient visited the emergency department prior to admission | Yes |
| ICU | If the patient was transferred to the ICU | Yes |
| SBP | Systolic Blood pressure | Yes |
| DBP | Diastolic Blood pressure | Yes |
| SaO2 | Oxygen saturation | Yes |
| Temperature | Body temperature | Yes |
| BMI | Body mass index | Yes |
| Weight | Weight of patients | Yes |
| DA04 | https://icd.who.int/browse10/2019/en https://medinfo.dk/sks/brows.php | Yes |
| DA41 | https://icd.who.int/browse10/2019/en https://medinfo.dk/sks/brows.php | Yes |
| DB18 | https://icd.who.int/browse10/2019/en https://medinfo.dk/sks/brows.php | Yes |
| DB25 | https://icd.who.int/browse10/2019/en https://medinfo.dk/sks/brows.php | Yes |
| DB37 | https://icd.who.int/browse10/2019/en https://medinfo.dk/sks/brows.php | Yes |
| DC22 | https://icd.who.int/browse10/2019/en https://medinfo.dk/sks/brows.php | Yes |
| DC25 | https://icd.who.int/browse10/2019/en https://medinfo.dk/sks/brows.php | Yes |
| DC71 | https://icd.who.int/browse10/2019/en https://medinfo.dk/sks/brows.php | Yes |
| DC78 | https://icd.who.int/browse10/2019/en https://medinfo.dk/sks/brows.php | Yes |
| DD17 | https://icd.who.int/browse10/2019/en https://medinfo.dk/sks/brows.php | Yes |
| DD33 | https://icd.who.int/browse10/2019/en https://medinfo.dk/sks/brows.php | Yes |
| DD43 | https://icd.who.int/browse10/2019/en https://medinfo.dk/sks/brows.php | Yes |
| DD50 | https://icd.who.int/browse10/2019/en https://medinfo.dk/sks/brows.php | Yes |
| DD64 | https://icd.who.int/browse10/2019/en https://medinfo.dk/sks/brows.php | Yes |
| DE41 | https://icd.who.int/browse10/2019/en https://medinfo.dk/sks/brows.php | Yes |
| DE63 | https://icd.who.int/browse10/2019/en https://medinfo.dk/sks/brows.php | Yes |
| DE64 | https://icd.who.int/browse10/2019/en https://medinfo.dk/sks/brows.php | Yes |
| DE78 | https://icd.who.int/browse10/2019/en https://medinfo.dk/sks/brows.php | Yes |
| DE83 | https://icd.who.int/browse10/2019/en https://medinfo.dk/sks/brows.php | Yes |
| DE86 | https://icd.who.int/browse10/2019/en https://medinfo.dk/sks/brows.php | Yes |
| DE87 | https://icd.who.int/browse10/2019/en https://medinfo.dk/sks/brows.php | Yes |
| DF10 | https://icd.who.int/browse10/2019/en https://medinfo.dk/sks/brows.php | Yes |
| DG20 | https://icd.who.int/browse10/2019/en https://medinfo.dk/sks/brows.php | Yes |
| DG25 | https://icd.who.int/browse10/2019/en https://medinfo.dk/sks/brows.php | Yes |
| DG35 | https://icd.who.int/browse10/2019/en https://medinfo.dk/sks/brows.php | Yes |
| DG40 | https://icd.who.int/browse10/2019/en https://medinfo.dk/sks/brows.php | Yes |
| DG43 | https://icd.who.int/browse10/2019/en https://medinfo.dk/sks/brows.php | Yes |
| DG45 | https://icd.who.int/browse10/2019/en https://medinfo.dk/sks/brows.php | Yes |
| DG56 | https://icd.who.int/browse10/2019/en https://medinfo.dk/sks/brows.php | Yes |
| DG57 | https://icd.who.int/browse10/2019/en https://medinfo.dk/sks/brows.php | Yes |
| DG61 | https://icd.who.int/browse10/2019/en https://medinfo.dk/sks/brows.php | Yes |
| DG62 | https://icd.who.int/browse10/2019/en https://medinfo.dk/sks/brows.php | Yes |
| DG63 | https://icd.who.int/browse10/2019/en https://medinfo.dk/sks/brows.php | Yes |
| DG83 | https://icd.who.int/browse10/2019/en https://medinfo.dk/sks/brows.php | Yes |
| DH81 | https://icd.who.int/browse10/2019/en https://medinfo.dk/sks/brows.php | Yes |
| DI61 | https://icd.who.int/browse10/2019/en https://medinfo.dk/sks/brows.php | Yes |
| DI63 | https://icd.who.int/browse10/2019/en https://medinfo.dk/sks/brows.php | Yes |
| DI65 | https://icd.who.int/browse10/2019/en https://medinfo.dk/sks/brows.php | Yes |
| DI69 | https://icd.who.int/browse10/2019/en https://medinfo.dk/sks/brows.php | Yes |
| DI70 | https://icd.who.int/browse10/2019/en https://medinfo.dk/sks/brows.php | Yes |
| DI85 | https://icd.who.int/browse10/2019/en https://medinfo.dk/sks/brows.php | Yes |
| DI86 | https://icd.who.int/browse10/2019/en https://medinfo.dk/sks/brows.php | Yes |
| DJ15 | https://icd.who.int/browse10/2019/en https://medinfo.dk/sks/brows.php | Yes |
| DJ86 | https://icd.who.int/browse10/2019/en https://medinfo.dk/sks/brows.php | Yes |
| DK20 | https://icd.who.int/browse10/2019/en https://medinfo.dk/sks/brows.php | Yes |
| DK21 | https://icd.who.int/browse10/2019/en https://medinfo.dk/sks/brows.php | Yes |
| DK22 | https://icd.who.int/browse10/2019/en https://medinfo.dk/sks/brows.php | Yes |
| DK25 | https://icd.who.int/browse10/2019/en https://medinfo.dk/sks/brows.php | Yes |
| DK26 | https://icd.who.int/browse10/2019/en https://medinfo.dk/sks/brows.php | Yes |
| DK29 | https://icd.who.int/browse10/2019/en https://medinfo.dk/sks/brows.php | Yes |
| DK30 | https://icd.who.int/browse10/2019/en https://medinfo.dk/sks/brows.php | Yes |
| DK44 | https://icd.who.int/browse10/2019/en https://medinfo.dk/sks/brows.php | Yes |
| DK50 | https://icd.who.int/browse10/2019/en https://medinfo.dk/sks/brows.php | Yes |
| DK51 | https://icd.who.int/browse10/2019/en https://medinfo.dk/sks/brows.php | Yes |
| DK52 | https://icd.who.int/browse10/2019/en https://medinfo.dk/sks/brows.php | Yes |
| DK57 | https://icd.who.int/browse10/2019/en https://medinfo.dk/sks/brows.php | Yes |
| DK58 | https://icd.who.int/browse10/2019/en https://medinfo.dk/sks/brows.php | Yes |
| DK59 | https://icd.who.int/browse10/2019/en https://medinfo.dk/sks/brows.php | Yes |
| DK65 | https://icd.who.int/browse10/2019/en https://medinfo.dk/sks/brows.php | Yes |
| DK70 | https://icd.who.int/browse10/2019/en https://medinfo.dk/sks/brows.php | Yes |
| DK71 | https://icd.who.int/browse10/2019/en https://medinfo.dk/sks/brows.php | Yes |
| DK72 | https://icd.who.int/browse10/2019/en https://medinfo.dk/sks/brows.php | Yes |
| DK74 | https://icd.who.int/browse10/2019/en https://medinfo.dk/sks/brows.php | Yes |
| DK76 | https://icd.who.int/browse10/2019/en https://medinfo.dk/sks/brows.php | Yes |
| DK80 | https://icd.who.int/browse10/2019/en https://medinfo.dk/sks/brows.php | Yes |
| DK81 | https://icd.who.int/browse10/2019/en https://medinfo.dk/sks/brows.php | Yes |
| DK83 | https://icd.who.int/browse10/2019/en https://medinfo.dk/sks/brows.php | Yes |
| DK85 | https://icd.who.int/browse10/2019/en https://medinfo.dk/sks/brows.php | Yes |
| DK86 | https://icd.who.int/browse10/2019/en https://medinfo.dk/sks/brows.php | Yes |
| DK90 | https://icd.who.int/browse10/2019/en https://medinfo.dk/sks/brows.php | Yes |
| DK91 | https://icd.who.int/browse10/2019/en https://medinfo.dk/sks/brows.php | Yes |
| DK92 | https://icd.who.int/browse10/2019/en https://medinfo.dk/sks/brows.php | Yes |
| DL02 | https://icd.who.int/browse10/2019/en https://medinfo.dk/sks/brows.php | Yes |
| DL03 | https://icd.who.int/browse10/2019/en https://medinfo.dk/sks/brows.php | Yes |
| DL04 | https://icd.who.int/browse10/2019/en https://medinfo.dk/sks/brows.php | Yes |
| DL08 | https://icd.who.int/browse10/2019/en https://medinfo.dk/sks/brows.php | Yes |
| DL92 | https://icd.who.int/browse10/2019/en https://medinfo.dk/sks/brows.php | Yes |
| DL98 | https://icd.who.int/browse10/2019/en https://medinfo.dk/sks/brows.php | Yes |
| DM00 | https://icd.who.int/browse10/2019/en https://medinfo.dk/sks/brows.php | Yes |
| DM16 | https://icd.who.int/browse10/2019/en https://medinfo.dk/sks/brows.php | Yes |
| DM17 | https://icd.who.int/browse10/2019/en https://medinfo.dk/sks/brows.php | Yes |
| DM18 | https://icd.who.int/browse10/2019/en https://medinfo.dk/sks/brows.php | Yes |
| DM19 | https://icd.who.int/browse10/2019/en https://medinfo.dk/sks/brows.php | Yes |
| DM20 | https://icd.who.int/browse10/2019/en https://medinfo.dk/sks/brows.php | Yes |
| DM21 | https://icd.who.int/browse10/2019/en https://medinfo.dk/sks/brows.php | Yes |
| DM23 | https://icd.who.int/browse10/2019/en https://medinfo.dk/sks/brows.php | Yes |
| DM24 | https://icd.who.int/browse10/2019/en https://medinfo.dk/sks/brows.php | Yes |
| DM25 | https://icd.who.int/browse10/2019/en https://medinfo.dk/sks/brows.php | Yes |
| DM35 | https://icd.who.int/browse10/2019/en https://medinfo.dk/sks/brows.php | Yes |
| DM47 | https://icd.who.int/browse10/2019/en https://medinfo.dk/sks/brows.php | Yes |
| DM48 | https://icd.who.int/browse10/2019/en https://medinfo.dk/sks/brows.php | Yes |
| DM54 | https://icd.who.int/browse10/2019/en https://medinfo.dk/sks/brows.php | Yes |
| DM65 | https://icd.who.int/browse10/2019/en https://medinfo.dk/sks/brows.php | Yes |
| DM70 | https://icd.who.int/browse10/2019/en https://medinfo.dk/sks/brows.php | Yes |
| DM75 | https://icd.who.int/browse10/2019/en https://medinfo.dk/sks/brows.php | Yes |
| DM79 | https://icd.who.int/browse10/2019/en https://medinfo.dk/sks/brows.php | Yes |
| DM84 | https://icd.who.int/browse10/2019/en https://medinfo.dk/sks/brows.php | Yes |
| DM87 | https://icd.who.int/browse10/2019/en https://medinfo.dk/sks/brows.php | Yes |
| DM93 | https://icd.who.int/browse10/2019/en https://medinfo.dk/sks/brows.php | Yes |
| DQ65 | https://icd.who.int/browse10/2019/en https://medinfo.dk/sks/brows.php | Yes |
| DQ66 | https://icd.who.int/browse10/2019/en https://medinfo.dk/sks/brows.php | Yes |
| DR10 | https://icd.who.int/browse10/2019/en https://medinfo.dk/sks/brows.php | Yes |
| DR18 | https://icd.who.int/browse10/2019/en https://medinfo.dk/sks/brows.php | Yes |
| DR19 | https://icd.who.int/browse10/2019/en https://medinfo.dk/sks/brows.php | Yes |
| DR25 | https://icd.who.int/browse10/2019/en https://medinfo.dk/sks/brows.php | Yes |
| DR29 | https://icd.who.int/browse10/2019/en https://medinfo.dk/sks/brows.php | Yes |
| DR42 | https://icd.who.int/browse10/2019/en https://medinfo.dk/sks/brows.php | Yes |
| DR51 | https://icd.who.int/browse10/2019/en https://medinfo.dk/sks/brows.php | Yes |
| DR56 | https://icd.who.int/browse10/2019/en https://medinfo.dk/sks/brows.php | Yes |
| DR60 | https://icd.who.int/browse10/2019/en https://medinfo.dk/sks/brows.php | Yes |
| DR63 | https://icd.who.int/browse10/2019/en https://medinfo.dk/sks/brows.php | Yes |
| DS06 | https://icd.who.int/browse10/2019/en https://medinfo.dk/sks/brows.php | Yes |
| DS22 | https://icd.who.int/browse10/2019/en https://medinfo.dk/sks/brows.php | Yes |
| DS32 | https://icd.who.int/browse10/2019/en https://medinfo.dk/sks/brows.php | Yes |
| DS40 | https://icd.who.int/browse10/2019/en https://medinfo.dk/sks/brows.php | Yes |
| DS42 | https://icd.who.int/browse10/2019/en https://medinfo.dk/sks/brows.php | Yes |
| DS43 | https://icd.who.int/browse10/2019/en https://medinfo.dk/sks/brows.php | Yes |
| DS44 | https://icd.who.int/browse10/2019/en https://medinfo.dk/sks/brows.php | Yes |
| DS46 | https://icd.who.int/browse10/2019/en https://medinfo.dk/sks/brows.php | Yes |
| DS51 | https://icd.who.int/browse10/2019/en https://medinfo.dk/sks/brows.php | Yes |
| DS52 | https://icd.who.int/browse10/2019/en https://medinfo.dk/sks/brows.php | Yes |
| DS53 | https://icd.who.int/browse10/2019/en https://medinfo.dk/sks/brows.php | Yes |
| DS54 | https://icd.who.int/browse10/2019/en https://medinfo.dk/sks/brows.php | Yes |
| DS60 | https://icd.who.int/browse10/2019/en https://medinfo.dk/sks/brows.php | Yes |
| DS61 | https://icd.who.int/browse10/2019/en https://medinfo.dk/sks/brows.php | Yes |
| DS62 | https://icd.who.int/browse10/2019/en https://medinfo.dk/sks/brows.php | Yes |
| DS63 | https://icd.who.int/browse10/2019/en https://medinfo.dk/sks/brows.php | Yes |
| DS66 | https://icd.who.int/browse10/2019/en https://medinfo.dk/sks/brows.php | Yes |
| DS70 | https://icd.who.int/browse10/2019/en https://medinfo.dk/sks/brows.php | Yes |
| DS72 | https://icd.who.int/browse10/2019/en https://medinfo.dk/sks/brows.php | Yes |
| DS80 | https://icd.who.int/browse10/2019/en https://medinfo.dk/sks/brows.php | Yes |
| DS81 | https://icd.who.int/browse10/2019/en https://medinfo.dk/sks/brows.php | Yes |
| DS82 | https://icd.who.int/browse10/2019/en https://medinfo.dk/sks/brows.php | Yes |
| DS83 | https://icd.who.int/browse10/2019/en https://medinfo.dk/sks/brows.php | Yes |
| DS91 | https://icd.who.int/browse10/2019/en https://medinfo.dk/sks/brows.php | Yes |
| DS92 | https://icd.who.int/browse10/2019/en https://medinfo.dk/sks/brows.php | Yes |
| DS93 | https://icd.who.int/browse10/2019/en https://medinfo.dk/sks/brows.php | Yes |
| DT07 | https://icd.who.int/browse10/2019/en https://medinfo.dk/sks/brows.php | Yes |
| DT12 | https://icd.who.int/browse10/2019/en https://medinfo.dk/sks/brows.php | Yes |
| DT14 | https://icd.who.int/browse10/2019/en https://medinfo.dk/sks/brows.php | Yes |
| DT39 | https://icd.who.int/browse10/2019/en https://medinfo.dk/sks/brows.php | Yes |
| DT84 | https://icd.who.int/browse10/2019/en https://medinfo.dk/sks/brows.php | Yes |
| DT87 | https://icd.who.int/browse10/2019/en https://medinfo.dk/sks/brows.php | Yes |
| DT88 | https://icd.who.int/browse10/2019/en https://medinfo.dk/sks/brows.php | Yes |
| DT91 | https://icd.who.int/browse10/2019/en https://medinfo.dk/sks/brows.php | Yes |
| DT92 | https://icd.who.int/browse10/2019/en https://medinfo.dk/sks/brows.php | Yes |
| DT93 | https://icd.who.int/browse10/2019/en https://medinfo.dk/sks/brows.php | Yes |
| DZ00 | https://icd.who.int/browse10/2019/en https://medinfo.dk/sks/brows.php | Yes |
| DZ03 | https://icd.who.int/browse10/2019/en https://medinfo.dk/sks/brows.php | Yes |
| DZ04 | https://icd.who.int/browse10/2019/en https://medinfo.dk/sks/brows.php | Yes |
| DZ50 | https://icd.who.int/browse10/2019/en https://medinfo.dk/sks/brows.php | Yes |
| DA09 | https://icd.who.int/browse10/2019/en https://medinfo.dk/sks/brows.php | No |
| DA26 | https://icd.who.int/browse10/2019/en https://medinfo.dk/sks/brows.php | No |
| DA35 | https://icd.who.int/browse10/2019/en https://medinfo.dk/sks/brows.php | No |
| DA46 | https://icd.who.int/browse10/2019/en https://medinfo.dk/sks/brows.php | No |
| DA49 | https://icd.who.int/browse10/2019/en https://medinfo.dk/sks/brows.php | No |
| DA87 | https://icd.who.int/browse10/2019/en https://medinfo.dk/sks/brows.php | No |
| DB27 | https://icd.who.int/browse10/2019/en https://medinfo.dk/sks/brows.php | No |
| DB95 | https://icd.who.int/browse10/2019/en https://medinfo.dk/sks/brows.php | No |
| DB99 | https://icd.who.int/browse10/2019/en https://medinfo.dk/sks/brows.php | No |
| DC15 | https://icd.who.int/browse10/2019/en https://medinfo.dk/sks/brows.php | No |
| DC16 | https://icd.who.int/browse10/2019/en https://medinfo.dk/sks/brows.php | No |
| DC17 | https://icd.who.int/browse10/2019/en https://medinfo.dk/sks/brows.php | No |
| DC24 | https://icd.who.int/browse10/2019/en https://medinfo.dk/sks/brows.php | No |
| DC34 | https://icd.who.int/browse10/2019/en https://medinfo.dk/sks/brows.php | No |
| DC40 | https://icd.who.int/browse10/2019/en https://medinfo.dk/sks/brows.php | No |
| DC44 | https://icd.who.int/browse10/2019/en https://medinfo.dk/sks/brows.php | No |
| DC45 | https://icd.who.int/browse10/2019/en https://medinfo.dk/sks/brows.php | No |
| DC49 | https://icd.who.int/browse10/2019/en https://medinfo.dk/sks/brows.php | No |
| DC76 | https://icd.who.int/browse10/2019/en https://medinfo.dk/sks/brows.php | No |
| DC79 | https://icd.who.int/browse10/2019/en https://medinfo.dk/sks/brows.php | No |
| DC80 | https://icd.who.int/browse10/2019/en https://medinfo.dk/sks/brows.php | No |
| DC83 | https://icd.who.int/browse10/2019/en https://medinfo.dk/sks/brows.php | No |
| DD12 | https://icd.who.int/browse10/2019/en https://medinfo.dk/sks/brows.php | No |
| DD13 | https://icd.who.int/browse10/2019/en https://medinfo.dk/sks/brows.php | No |
| DD16 | https://icd.who.int/browse10/2019/en https://medinfo.dk/sks/brows.php | No |
| DD21 | https://icd.who.int/browse10/2019/en https://medinfo.dk/sks/brows.php | No |
| DD32 | https://icd.who.int/browse10/2019/en https://medinfo.dk/sks/brows.php | No |
| DD35 | https://icd.who.int/browse10/2019/en https://medinfo.dk/sks/brows.php | No |
| DD37 | https://icd.who.int/browse10/2019/en https://medinfo.dk/sks/brows.php | No |
| DD55 | https://icd.who.int/browse10/2019/en https://medinfo.dk/sks/brows.php | No |
| DD61 | https://icd.who.int/browse10/2019/en https://medinfo.dk/sks/brows.php | No |
| DD68 | https://icd.who.int/browse10/2019/en https://medinfo.dk/sks/brows.php | No |
| DE10 | https://icd.who.int/browse10/2019/en https://medinfo.dk/sks/brows.php | No |
| DE11 | https://icd.who.int/browse10/2019/en https://medinfo.dk/sks/brows.php | No |
| DE13 | https://icd.who.int/browse10/2019/en https://medinfo.dk/sks/brows.php | No |
| DE14 | https://icd.who.int/browse10/2019/en https://medinfo.dk/sks/brows.php | No |
| DE16 | https://icd.who.int/browse10/2019/en https://medinfo.dk/sks/brows.php | No |
| DE23 | https://icd.who.int/browse10/2019/en https://medinfo.dk/sks/brows.php | No |
| DE34 | https://icd.who.int/browse10/2019/en https://medinfo.dk/sks/brows.php | No |
| DE46 | https://icd.who.int/browse10/2019/en https://medinfo.dk/sks/brows.php | No |
| DE51 | https://icd.who.int/browse10/2019/en https://medinfo.dk/sks/brows.php | No |
| DE61 | https://icd.who.int/browse10/2019/en https://medinfo.dk/sks/brows.php | No |
| DE65 | https://icd.who.int/browse10/2019/en https://medinfo.dk/sks/brows.php | No |
| DE66 | https://icd.who.int/browse10/2019/en https://medinfo.dk/sks/brows.php | No |
| DE73 | https://icd.who.int/browse10/2019/en https://medinfo.dk/sks/brows.php | No |
| DF00 | https://icd.who.int/browse10/2019/en https://medinfo.dk/sks/brows.php | No |
| DF01 | https://icd.who.int/browse10/2019/en https://medinfo.dk/sks/brows.php | No |
| DF03 | https://icd.who.int/browse10/2019/en https://medinfo.dk/sks/brows.php | No |
| DF06 | https://icd.who.int/browse10/2019/en https://medinfo.dk/sks/brows.php | No |
| DF44 | https://icd.who.int/browse10/2019/en https://medinfo.dk/sks/brows.php | No |
| DF45 | https://icd.who.int/browse10/2019/en https://medinfo.dk/sks/brows.php | No |
| DG03 | https://icd.who.int/browse10/2019/en https://medinfo.dk/sks/brows.php | No |
| DG04 | https://icd.who.int/browse10/2019/en https://medinfo.dk/sks/brows.php | No |
| DG06 | https://icd.who.int/browse10/2019/en https://medinfo.dk/sks/brows.php | No |
| DG11 | https://icd.who.int/browse10/2019/en https://medinfo.dk/sks/brows.php | No |
| DG12 | https://icd.who.int/browse10/2019/en https://medinfo.dk/sks/brows.php | No |
| DG13 | https://icd.who.int/browse10/2019/en https://medinfo.dk/sks/brows.php | No |
| DG23 | https://icd.who.int/browse10/2019/en https://medinfo.dk/sks/brows.php | No |
| DG24 | https://icd.who.int/browse10/2019/en https://medinfo.dk/sks/brows.php | No |
| DG31 | https://icd.who.int/browse10/2019/en https://medinfo.dk/sks/brows.php | No |
| DG36 | https://icd.who.int/browse10/2019/en https://medinfo.dk/sks/brows.php | No |
| DG37 | https://icd.who.int/browse10/2019/en https://medinfo.dk/sks/brows.php | No |
| DG41 | https://icd.who.int/browse10/2019/en https://medinfo.dk/sks/brows.php | No |
| DG44 | https://icd.who.int/browse10/2019/en https://medinfo.dk/sks/brows.php | No |
| DG50 | https://icd.who.int/browse10/2019/en https://medinfo.dk/sks/brows.php | No |
| DG51 | https://icd.who.int/browse10/2019/en https://medinfo.dk/sks/brows.php | No |
| DG52 | https://icd.who.int/browse10/2019/en https://medinfo.dk/sks/brows.php | No |
| DG54 | https://icd.who.int/browse10/2019/en https://medinfo.dk/sks/brows.php | No |
| DG58 | https://icd.who.int/browse10/2019/en https://medinfo.dk/sks/brows.php | No |
| DG60 | https://icd.who.int/browse10/2019/en https://medinfo.dk/sks/brows.php | No |
| DG70 | https://icd.who.int/browse10/2019/en https://medinfo.dk/sks/brows.php | No |
| DG71 | https://icd.who.int/browse10/2019/en https://medinfo.dk/sks/brows.php | No |
| DG73 | https://icd.who.int/browse10/2019/en https://medinfo.dk/sks/brows.php | No |
| DG80 | https://icd.who.int/browse10/2019/en https://medinfo.dk/sks/brows.php | No |
| DG82 | https://icd.who.int/browse10/2019/en https://medinfo.dk/sks/brows.php | No |
| DG90 | https://icd.who.int/browse10/2019/en https://medinfo.dk/sks/brows.php | No |
| DG93 | https://icd.who.int/browse10/2019/en https://medinfo.dk/sks/brows.php | No |
| DG95 | https://icd.who.int/browse10/2019/en https://medinfo.dk/sks/brows.php | No |
| DG96 | https://icd.who.int/browse10/2019/en https://medinfo.dk/sks/brows.php | No |
| DG97 | https://icd.who.int/browse10/2019/en https://medinfo.dk/sks/brows.php | No |
| DG98 | https://icd.who.int/browse10/2019/en https://medinfo.dk/sks/brows.php | No |
| DH34 | https://icd.who.int/browse10/2019/en https://medinfo.dk/sks/brows.php | No |
| DH46 | https://icd.who.int/browse10/2019/en https://medinfo.dk/sks/brows.php | No |
| DH49 | https://icd.who.int/browse10/2019/en https://medinfo.dk/sks/brows.php | No |
| DH53 | https://icd.who.int/browse10/2019/en https://medinfo.dk/sks/brows.php | No |
| DH57 | https://icd.who.int/browse10/2019/en https://medinfo.dk/sks/brows.php | No |
| DI21 | https://icd.who.int/browse10/2019/en https://medinfo.dk/sks/brows.php | No |
| DI26 | https://icd.who.int/browse10/2019/en https://medinfo.dk/sks/brows.php | No |
| DI38 | https://icd.who.int/browse10/2019/en https://medinfo.dk/sks/brows.php | No |
| DI48 | https://icd.who.int/browse10/2019/en https://medinfo.dk/sks/brows.php | No |
| DI60 | https://icd.who.int/browse10/2019/en https://medinfo.dk/sks/brows.php | No |
| DI64 | https://icd.who.int/browse10/2019/en https://medinfo.dk/sks/brows.php | No |
| DI67 | https://icd.who.int/browse10/2019/en https://medinfo.dk/sks/brows.php | No |
| DI68 | https://icd.who.int/browse10/2019/en https://medinfo.dk/sks/brows.php | No |
| DI73 | https://icd.who.int/browse10/2019/en https://medinfo.dk/sks/brows.php | No |
| DI74 | https://icd.who.int/browse10/2019/en https://medinfo.dk/sks/brows.php | No |
| DI78 | https://icd.who.int/browse10/2019/en https://medinfo.dk/sks/brows.php | No |
| DI81 | https://icd.who.int/browse10/2019/en https://medinfo.dk/sks/brows.php | No |
| DI84 | https://icd.who.int/browse10/2019/en https://medinfo.dk/sks/brows.php | No |
| DI89 | https://icd.who.int/browse10/2019/en https://medinfo.dk/sks/brows.php | No |
| DI95 | https://icd.who.int/browse10/2019/en https://medinfo.dk/sks/brows.php | No |
| DJ15 | https://icd.who.int/browse10/2019/en https://medinfo.dk/sks/brows.php | No |
| DJ18 | https://icd.who.int/browse10/2019/en https://medinfo.dk/sks/brows.php | No |
| DJ69 | https://icd.who.int/browse10/2019/en https://medinfo.dk/sks/brows.php | No |
| DJ86 | https://icd.who.int/browse10/2019/en https://medinfo.dk/sks/brows.php | No |
| DK27 | https://icd.who.int/browse10/2019/en https://medinfo.dk/sks/brows.php | No |
| DK28 | https://icd.who.int/browse10/2019/en https://medinfo.dk/sks/brows.php | No |
| DK31 | https://icd.who.int/browse10/2019/en https://medinfo.dk/sks/brows.php | No |
| DK42 | https://icd.who.int/browse10/2019/en https://medinfo.dk/sks/brows.php | No |
| DK55 | https://icd.who.int/browse10/2019/en https://medinfo.dk/sks/brows.php | No |
| DK56 | https://icd.who.int/browse10/2019/en https://medinfo.dk/sks/brows.php | No |
| DK62 | https://icd.who.int/browse10/2019/en https://medinfo.dk/sks/brows.php | No |
| DK63 | https://icd.who.int/browse10/2019/en https://medinfo.dk/sks/brows.php | No |
| DK75 | https://icd.who.int/browse10/2019/en https://medinfo.dk/sks/brows.php | No |
| DL60 | https://icd.who.int/browse10/2019/en https://medinfo.dk/sks/brows.php | No |
| DL70 | https://icd.who.int/browse10/2019/en https://medinfo.dk/sks/brows.php | No |
| DL97 | https://icd.who.int/browse10/2019/en https://medinfo.dk/sks/brows.php | No |
| DM02 | https://icd.who.int/browse10/2019/en https://medinfo.dk/sks/brows.php | No |
| DM05 | https://icd.who.int/browse10/2019/en https://medinfo.dk/sks/brows.php | No |
| DM06 | https://icd.who.int/browse10/2019/en https://medinfo.dk/sks/brows.php | No |
| DM07 | https://icd.who.int/browse10/2019/en https://medinfo.dk/sks/brows.php | No |
| DM11 | https://icd.who.int/browse10/2019/en https://medinfo.dk/sks/brows.php | No |
| DM12 | https://icd.who.int/browse10/2019/en https://medinfo.dk/sks/brows.php | No |
| DM13 | https://icd.who.int/browse10/2019/en https://medinfo.dk/sks/brows.php | No |
| DM15 | https://icd.who.int/browse10/2019/en https://medinfo.dk/sks/brows.php | No |
| DM22 | https://icd.who.int/browse10/2019/en https://medinfo.dk/sks/brows.php | No |
| DM36 | https://icd.who.int/browse10/2019/en https://medinfo.dk/sks/brows.php | No |
| DM41 | https://icd.who.int/browse10/2019/en https://medinfo.dk/sks/brows.php | No |
| DM43 | https://icd.who.int/browse10/2019/en https://medinfo.dk/sks/brows.php | No |
| DM46 | https://icd.who.int/browse10/2019/en https://medinfo.dk/sks/brows.php | No |
| DM50 | https://icd.who.int/browse10/2019/en https://medinfo.dk/sks/brows.php | No |
| DM51 | https://icd.who.int/browse10/2019/en https://medinfo.dk/sks/brows.php | No |
| DM53 | https://icd.who.int/browse10/2019/en https://medinfo.dk/sks/brows.php | No |
| DM60 | https://icd.who.int/browse10/2019/en https://medinfo.dk/sks/brows.php | No |
| DM61 | https://icd.who.int/browse10/2019/en https://medinfo.dk/sks/brows.php | No |
| DM66 | https://icd.who.int/browse10/2019/en https://medinfo.dk/sks/brows.php | No |
| DM67 | https://icd.who.int/browse10/2019/en https://medinfo.dk/sks/brows.php | No |
| DM71 | https://icd.who.int/browse10/2019/en https://medinfo.dk/sks/brows.php | No |
| DM72 | https://icd.who.int/browse10/2019/en https://medinfo.dk/sks/brows.php | No |
| DM76 | https://icd.who.int/browse10/2019/en https://medinfo.dk/sks/brows.php | No |
| DM77 | https://icd.who.int/browse10/2019/en https://medinfo.dk/sks/brows.php | No |
| DM80 | https://icd.who.int/browse10/2019/en https://medinfo.dk/sks/brows.php | No |
| DM83 | https://icd.who.int/browse10/2019/en https://medinfo.dk/sks/brows.php | No |
| DM85 | https://icd.who.int/browse10/2019/en https://medinfo.dk/sks/brows.php | No |
| DM86 | https://icd.who.int/browse10/2019/en https://medinfo.dk/sks/brows.php | No |
| DM89 | https://icd.who.int/browse10/2019/en https://medinfo.dk/sks/brows.php | No |
| DM91 | https://icd.who.int/browse10/2019/en https://medinfo.dk/sks/brows.php | No |
| DM95 | https://icd.who.int/browse10/2019/en https://medinfo.dk/sks/brows.php | No |
| DM96 | https://icd.who.int/browse10/2019/en https://medinfo.dk/sks/brows.php | No |
| DN30 | https://icd.who.int/browse10/2019/en https://medinfo.dk/sks/brows.php | No |
| DN39 | https://icd.who.int/browse10/2019/en https://medinfo.dk/sks/brows.php | No |
| DQ28 | https://icd.who.int/browse10/2019/en https://medinfo.dk/sks/brows.php | No |
| DQ61 | https://icd.who.int/browse10/2019/en https://medinfo.dk/sks/brows.php | No |
| DQ71 | https://icd.who.int/browse10/2019/en https://medinfo.dk/sks/brows.php | No |
| DQ78 | https://icd.who.int/browse10/2019/en https://medinfo.dk/sks/brows.php | No |
| DR02 | https://icd.who.int/browse10/2019/en https://medinfo.dk/sks/brows.php | No |
| DR07 | https://icd.who.int/browse10/2019/en https://medinfo.dk/sks/brows.php | No |
| DR11 | https://icd.who.int/browse10/2019/en https://medinfo.dk/sks/brows.php | No |
| DR13 | https://icd.who.int/browse10/2019/en https://medinfo.dk/sks/brows.php | No |
| DR17 | https://icd.who.int/browse10/2019/en https://medinfo.dk/sks/brows.php | No |
| DR20 | https://icd.who.int/browse10/2019/en https://medinfo.dk/sks/brows.php | No |
| DR21 | https://icd.who.int/browse10/2019/en https://medinfo.dk/sks/brows.php | No |
| DR26 | https://icd.who.int/browse10/2019/en https://medinfo.dk/sks/brows.php | No |
| DR41 | https://icd.who.int/browse10/2019/en https://medinfo.dk/sks/brows.php | No |
| DR50 | https://icd.who.int/browse10/2019/en https://medinfo.dk/sks/brows.php | No |
| DR52 | https://icd.who.int/browse10/2019/en https://medinfo.dk/sks/brows.php | No |
| DR55 | https://icd.who.int/browse10/2019/en https://medinfo.dk/sks/brows.php | No |
| DR74 | https://icd.who.int/browse10/2019/en https://medinfo.dk/sks/brows.php | No |
| DR90 | https://icd.who.int/browse10/2019/en https://medinfo.dk/sks/brows.php | No |
| DR93 | https://icd.who.int/browse10/2019/en https://medinfo.dk/sks/brows.php | No |
| DS00 | https://icd.who.int/browse10/2019/en https://medinfo.dk/sks/brows.php | No |
| DS01 | https://icd.who.int/browse10/2019/en https://medinfo.dk/sks/brows.php | No |
| DS02 | https://icd.who.int/browse10/2019/en https://medinfo.dk/sks/brows.php | No |
| DS04 | https://icd.who.int/browse10/2019/en https://medinfo.dk/sks/brows.php | No |
| DS12 | https://icd.who.int/browse10/2019/en https://medinfo.dk/sks/brows.php | No |
| DS14 | https://icd.who.int/browse10/2019/en https://medinfo.dk/sks/brows.php | No |
| DS30 | https://icd.who.int/browse10/2019/en https://medinfo.dk/sks/brows.php | No |
| DS33 | https://icd.who.int/browse10/2019/en https://medinfo.dk/sks/brows.php | No |
| DS34 | https://icd.who.int/browse10/2019/en https://medinfo.dk/sks/brows.php | No |
| DS50 | https://icd.who.int/browse10/2019/en https://medinfo.dk/sks/brows.php | No |
| DS67 | https://icd.who.int/browse10/2019/en https://medinfo.dk/sks/brows.php | No |
| DS68 | https://icd.who.int/browse10/2019/en https://medinfo.dk/sks/brows.php | No |
| DS69 | https://icd.who.int/browse10/2019/en https://medinfo.dk/sks/brows.php | No |
| DS71 | https://icd.who.int/browse10/2019/en https://medinfo.dk/sks/brows.php | No |
| DS73 | https://icd.who.int/browse10/2019/en https://medinfo.dk/sks/brows.php | No |
| DS76 | https://icd.who.int/browse10/2019/en https://medinfo.dk/sks/brows.php | No |
| DS79 | https://icd.who.int/browse10/2019/en https://medinfo.dk/sks/brows.php | No |
| DS80 | https://icd.who.int/browse10/2019/en https://medinfo.dk/sks/brows.php | No |
| DS86 | https://icd.who.int/browse10/2019/en https://medinfo.dk/sks/brows.php | No |
| DS90 | https://icd.who.int/browse10/2019/en https://medinfo.dk/sks/brows.php | No |
| DT42 | https://icd.who.int/browse10/2019/en https://medinfo.dk/sks/brows.php | No |
| DT78 | https://icd.who.int/browse10/2019/en https://medinfo.dk/sks/brows.php | No |
| DT79 | https://icd.who.int/browse10/2019/en https://medinfo.dk/sks/brows.php | No |
| DT80 | https://icd.who.int/browse10/2019/en https://medinfo.dk/sks/brows.php | No |
| DT81 | https://icd.who.int/browse10/2019/en https://medinfo.dk/sks/brows.php | No |
| DT82 | https://icd.who.int/browse10/2019/en https://medinfo.dk/sks/brows.php | No |
| DT85 | https://icd.who.int/browse10/2019/en https://medinfo.dk/sks/brows.php | No |
| DT86 | https://icd.who.int/browse10/2019/en https://medinfo.dk/sks/brows.php | No |
| DT98 | https://icd.who.int/browse10/2019/en https://medinfo.dk/sks/brows.php | No |
| DZ02 | https://icd.who.int/browse10/2019/en https://medinfo.dk/sks/brows.php | No |
| DZ09 | https://icd.who.int/browse10/2019/en https://medinfo.dk/sks/brows.php | No |
| DZ88 | https://icd.who.int/browse10/2019/en https://medinfo.dk/sks/brows.php | No |
| DZ90 | https://icd.who.int/browse10/2019/en https://medinfo.dk/sks/brows.php | No |
| DZ94 | https://icd.who.int/browse10/2019/en https://medinfo.dk/sks/brows.php | No |
