# Supplementary material for: AUD-DSS: a decision support system for early detection of patients with alcohol use disorder
Source: BMC Bioinformatics. 2023 Sep 2;24:329. doi: 10.1186/s12859-023-05450-6 (PMC10474761; doi:10.1186/s12859-023-05450-6)
Supplement: Supplementary file 4 — Additional file 4. Average performance of the developed models based on test set and validation set. [file 12859_2023_5450_MOESM4_ESM.docx]

| ***Table 1. Average performance of the developed models based on test set*** | | | | | | |
| --- | --- | --- | --- | --- | --- | --- |
| **Model** | **Precision**  **(Positive Predictive Value)** | **Recall**  **(Sensitivity)** | **F1-score** | **Accuracy** | **AUROC** | **AUPRC** |
| **Baseline** | | | | | | |
| **Stacking Ensemble**  **(AUDPM)** | 0.91 | 0.78 | 0.83 | 0.92 | 0.95 | 0.70 |
| **Random Forest** | 0.94 | 0.70 | 0.75 | 0.90 | 0.95 | 0.56 |
| **Decision Tree** | 0.74 | 0.72 | 0.73 | 0.85 | 0.76 | 0.54 |
| **K-Nearest Neighbour** | 0.78 | 0.76 | 0.77 | 0.88 | 0.84 | 0.61 |
| **Support Vector Machine** | 0.90 | 0.62 | 0.66 | 0.87 | 0.86 | 0.38 |
| **XGBoost** | 0.87 | 0.73 | 0.77 | 0.90 | 0.73 | 0.56 |
| **Proposed Pipeline** | | | | | | |
| **Stacking Ensemble**  **(AUDPM)** | 0.97 | 0.96 | 0.97 | 0.98 | 0.99 | 0.90 |
| **Random Forest** | 0.97 | 0.89 | 0.93 | 0.96 | 0.99 | 0.87 |
| **Decision Tree** | 0.87 | 0.80 | 0.83 | 0.91 | 0.91 | 0.70 |
| **K-Nearest Neighbour** | 0.79 | 0.74 | 0.76 | 0.88 | 0.86 | 0.59 |
| **Support Vector Machine** | 0.96 | 0.81 | 0.86 | 0.93 | 0.95 | 0.75 |
| **XGBoost** | 0.90 | 0.73 | 0.78 | 0.90 | 0.73 | 0.62 |

| ***Table 2. Average performance of the developed models based on validation set*** | | | | | | |
| --- | --- | --- | --- | --- | --- | --- |
| **Model** | **Precision**  **(Positive Predictive Value)** | **Recall**  **(Sensitivity)** | **F1-score** | **Accuracy** | **AUROC** | **AUPRC** |
| **Baseline** | | | | | | |
| **Stacking Ensemble**  **(AUDPM)** | 0.98 | 0.98 | 0.98 | 0.98 | 0.98 | 0.92 |
| **Random Forest** | 0.98 | 0.91 | 0.94 | 0.97 | 0.99 | 0.89 |
| **Decision Tree** | 0.94 | 0.94 | 0.94 | 0.94 | 0.93 | 0.81 |
| **K-Nearest Neighbour** | 0.85 | 0.82 | 0.83 | 0.91 | 0.89 | 0.72 |
| **Support Vector Machine** | 0.96 | 0.82 | 0.88 | 0.94 | 0.95 | 0.79 |
| **XGBoost** | 0.96 | 0.91 | 0.94 | 0.97 | 0.97 | 0.89 |
| **Proposed Pipeline** | | | | | | |
| **Stacking Ensemble**  **(AUDPM)** | 0.98 | 0.98 | 0.98 | 0.98 | 0.99 | 0.95 |
| **Random Forest** | 0.99 | 0.97 | 0.97 | 0.97 | 0.99 | 0.92 |
| **Decision Tree** | 0.92 | 0.92 | 0.92 | 0.92 | 0.94 | 0.84 |
| **K-Nearest Neighbour** | 0.87 | 0.88 | 0.88 | 0.89 | 0.87 | 0.76 |
| **Support Vector Machine** | 0.96 | 0.81 | 0.86 | 0.93 | 0.95 | 0.76 |
| **XGBoost** | 0.90 | 0.90 | 0.89 | 0.90 | 0.90 | 0.79 |
